# Single-cell multi-omics in the medicinal plant *Catharanthus roseus*

**DOI:** 10.1038/s41589-023-01327-0

**Published:** 2023-05-15

**Authors:** Chenxin Li, Joshua C. Wood, Anh Hai Vu, John P. Hamilton, Carlos Eduardo Rodriguez Lopez, Richard M. E. Payne, Delia Ayled Serna Guerrero, Klaus Gase, Kotaro Yamamoto, Brieanne Vaillancourt, Lorenzo Caputi, Sarah E. O’Connor, C. Robin Buell

**Affiliations:** 1 Center for Applied Genetic Technologies, University of Georgia, Athens, GA USA; 2 Department of Natural Product Biosynthesis, Max Planck Institute for Chemical Ecology, Jena, Germany; 3 Escuela de Ingenieria y Ciencias, Tecnologico de Monterrey, Monterrey, Mexico; 4 The John Innes Centre, Department of Biological Chemistry, Norwich Research Park, Norwich, UK; 5 School of Science, Association of International Arts and Science, Yokohama City University, Yokohama, Japan; 6 Department of Crop and Soil Sciences, University of Georgia, Athens, GA USA; 7 Institute of Plant Breeding, Genetics and Genomics, University of Georgia, Athens, GA USA

**Keywords:** Plant sciences, Biological techniques, Biochemistry

## Abstract

Advances in omics technologies now permit the generation of highly contiguous genome assemblies, detection of transcripts and metabolites at the level of single cells and high-resolution determination of gene regulatory features. Here, using a complementary, multi-omics approach, we interrogated the monoterpene indole alkaloid (MIA) biosynthetic pathway in *Catharanthus roseus*, a source of leading anticancer drugs. We identified clusters of genes involved in MIA biosynthesis on the eight *C. roseus* chromosomes and extensive gene duplication of MIA pathway genes. Clustering was not limited to the linear genome, and through chromatin interaction data, MIA pathway genes were present within the same topologically associated domain, permitting the identification of a secologanin transporter. Single-cell RNA-sequencing revealed sequential cell-type-specific partitioning of the leaf MIA biosynthetic pathway that, when coupled with a single-cell metabolomics approach, permitted the identification of a reductase that yields the bis-indole alkaloid anhydrovinblastine. We also revealed cell-type-specific expression in the root MIA pathway.

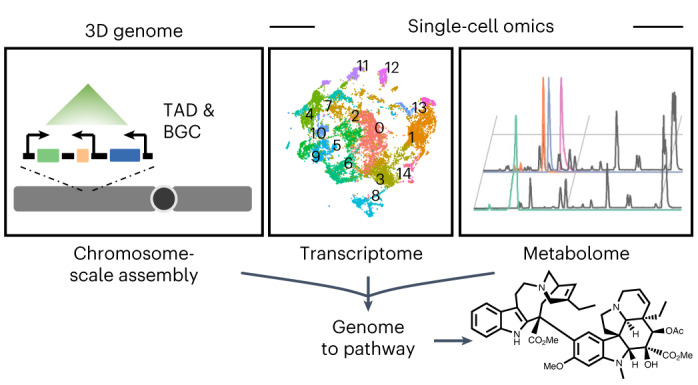

## Abstract (web summary)

The medicinal plant *Catharanthus roseus* is a source of leading anticancer drugs. The monoterpene indole alkaloid (MIA) biosynthetic pathway in *C. roseus* has now been analyzed using a complementary, multi-omics, single-cell approach. This identified clusters of genes involved in MIA biosynthesis and cell-type-specific partitioning in the MIA biosynthetic pathway.

## Main

Gene discovery for metabolic pathways in plants has relied on whole-tissue-derived datasets^1^. Discovery entails correlating the expression of genes with the presence of the molecule of interest. Occasionally, high-quality genome assemblies further facilitate pathway gene discovery by allowing the identification of biosynthetic gene clusters, but such clusters occur in limited numbers of plant pathways. Overall, identifying all genes in a complex metabolic pathway typically requires functional screening of large numbers of candidate genes; these mining approaches are further limited when genes are not coregulated.

**Fig. 1:**
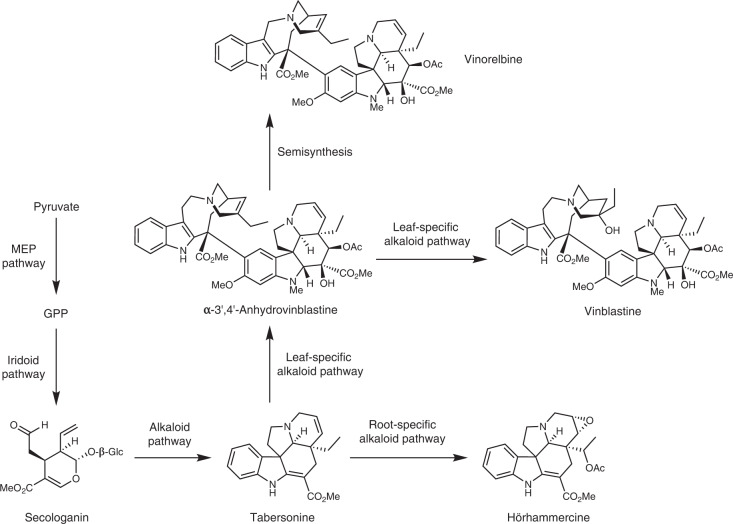
Abbreviated biosynthetic pathway of monoterpene indole alkaloids in *Catharanthus roseus*. For a more extended biosynthetic scheme, see Supplementary Fig. 1 (leaves) and Supplementary Fig. 2 (roots).

In plants, biosynthetic pathways of these complex specialized metabolites (natural products) are localized not only to distinct organs but also to distinct cell types within organs. The advent of single-cell omics has enormous potential to revolutionize metabolic pathway gene discovery in plants^2–4^. Furthermore, single-cell omics reveal how metabolic pathways are partitioned across cell types. The medicinal plant species *Catharanthus roseus* (L.) G. Don produces monoterpene indole alkaloids (MIAs), a natural product family with a wide variety of chemical scaffolds and biological activities^5^. These include the dimeric MIAs that demonstrate anticancer activity (vinblastine and vincristine) or are used as a precursor (anhydrovinblastine) for alkaloids with anticancer activity (for example, vinorelbine; Fig. 1 and Supplementary Fig. 1). MIA biosynthesis in *C. roseus* shows distinct metabolite profiles across organs, with leaves producing vinblastine and vincristine and roots producing hörhammercine (Fig. 1 and Supplementary Fig 2). Over the last 30 years, 38 dedicated MIA pathway genes and several transcription factors involved in the jasmonate-induction of the MIA biosynthetic pathway have been discovered using traditional biochemical and coexpression analysis of whole-tissue-derived omics datasets. Not only does *C. roseus* have enormous economic importance as a producer of anticancer drugs, but it has also emerged as a model species to probe the mechanistic basis of localization, transport and regulation of specialized metabolic pathways.

Here we show how a state-of-the-art genome assembly, Hi-C chromosome conformation capture and single-cell transcriptomics datasets empowered discoveries in the *C. roseus* MIA biosynthetic pathway. We show that a 38-step MIA pathway is sequentially expressed in three distinct cell types in leaves, and also show differences in cell-type-specific gene expression of the MIA biosynthetic pathway in *C. roseus* roots. We used long-range chromosome interaction maps to reveal the three-dimensional (3D) organization of MIA biosynthetic gene clusters that contribute to coordinated gene expression. To complement our genomic and transcriptomic datasets, we developed a high-throughput, high-resolution and semi-quantitative single-cell metabolomics (scMet) profiling method for *C. roseus* leaf cells. Finally, using these omics data, we identified an intracellular transporter and the missing reductase that generates anhydrovinblastine.

## Results

### A chromosome-scale genome assembly for *C. roseus*

**Fig. 2:**
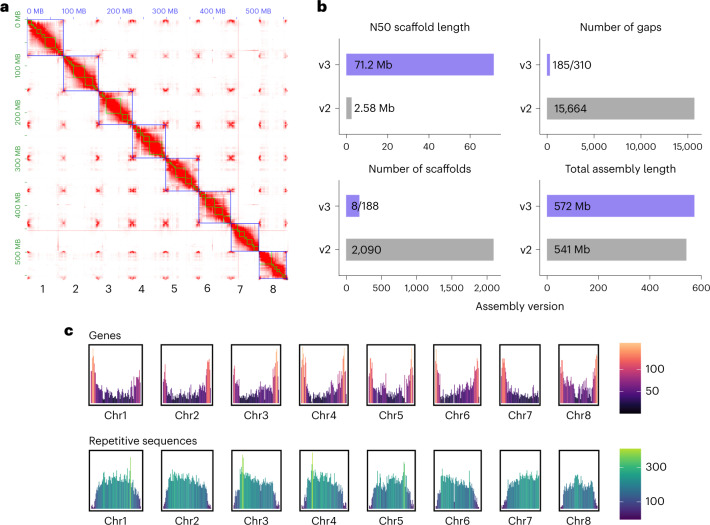
Chromosome-scale assembly and annotation of *Catharanthus roseus*. **a**, Contact map of Hi-C reads revealing the eight chromosomes of *C. roseus*. Blue boxes represent pseudomolecules, green boxes represent contigs of the primary assembly and red color indicates Hi-C contacts. **b**, Assembly metrics of the v3 versus v2 *C. roseus* genome assembly. For the number of gaps and scaffolds, the v3 numbers represent the pseudochromosomes and the complete assembly (pseudochromosomes plus unanchored scaffolds). **c**, Gene and repetitive sequence density along the eight *C. roseus* chromosomes. *Y* axis values and color scales represent the number of representative gene models (first row) or repetitive sequences (>1-kb, second row) in 1-Mb resolution. Source data

Because some specialized metabolic pathways have been demonstrated to be physically clustered in plant genomes, the availability of a scaffolded genome is essential to accelerate gene discovery^6^. Earlier versions of the *C. roseus* genome assembly were fragmented^7^. Here using Oxford Nanopore Technologies (ONT) long reads, we generated a draft assembly for *C. roseus* ‘Sunstorm Apricot’ and performed error correction using ONT and Illumina whole-genome shotgun reads yielding a 575.2 Mb assembly composed of 1,411 contigs with an N50 contig length of 11.3 Mb. Proximity-by-ligation Hi-C sequences were used to scaffold the contigs, resulting in eight pseudochromosomes (Fig. 2a), consistent with the chromosome number of *C. roseus*. To fill gaps within the pseudochromosomes, we capitalized on the ability to redirect in real-time the sequencing of each nanopore using adaptive sampling^8^ by targeting the physical ends of each contig for sequencing (Supplementary Fig. 3; adaptive finishing). We observed 5.5- to 14-fold enrichment of sequence coverage, depending on the length of physical ends that we targeted (Supplementary Fig. 3a). Using the adaptive finishing reads along with the bulk ONT genomic reads, we closed 14 gaps ranging in size from 8-bp to 20.2-kbp (Supplementary Fig. 3b and Supplementary Table 1). The final (v3) *C. roseus* genome assembly is 572.1 Mb, of which 556.4 Mb is anchored to the eight chromosomes, with an N50 scaffold size of 71.2 Mb (Fig. 2b) representing a substantial improvement in contiguity (27.6-fold increase in N50 scaffold length) and 31 Mb genome size increase over v2 of the *C. roseus* genome^7^. Assessment of the v3 *C. roseus* genome using Benchmarking Universal Single-Copy Ortholog (BUSCO) analysis revealed 98.5% BUSCOs indicating a high-quality genome assembly (Supplementary Table 2).

To annotate the genome, we performed de novo repeat identification, revealing 70.25% of the genome was repetitive with retroelements being the largest class of transposable elements (Supplementary Table 3). Genome-guided transcript assemblies from paired-end mRNA-sequencing (mRNA-seq) reads from diverse tissues (leaf, root, flower, shoots, methyl jasmonate treatment; Supplementary Table 4) were used to train an ab initio gene finder and generate primary gene models. We generated 62 million ONT full-length cDNA (FL-cDNA) reads (Supplementary Table 4) and used these data, along with the mRNA-seq data, to refine our gene model structures. The final annotated gene set encompasses 26,347 genes encoding 66,262 gene models (Supplementary Table 5) with an average of three alternative splice forms per locus, attributable to the deep transcript data used in the annotation. BUSCO analysis of the annotated gene models revealed 96.1% complete BUSCOs (Supplementary Table 2), suggestive of high-quality annotation that was confirmed by manual inspection and curation of known MIA pathway genes (Supplementary Table 6).

**Fig. 3:**
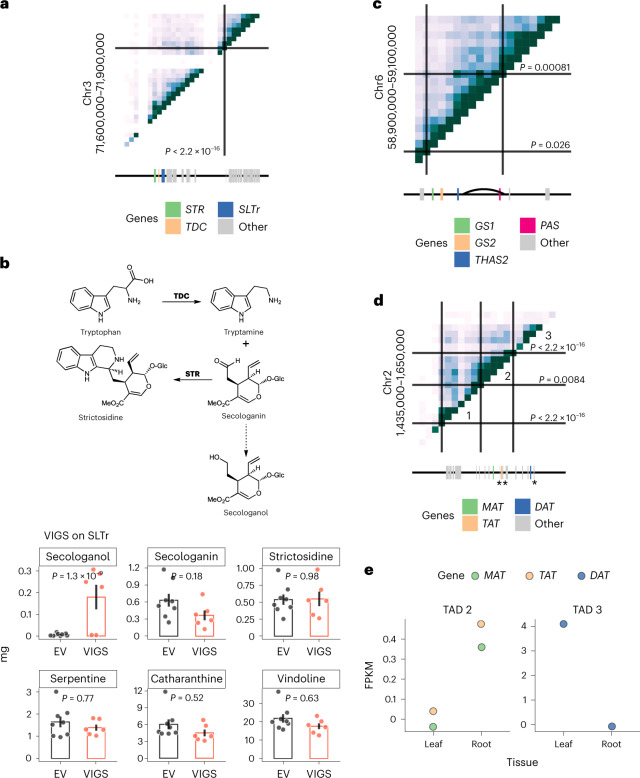
Biosynthetic gene clusters and associated 3D chromosome features. **a**, Hi-C contact map generated from mature leaves for a gene cluster consisting of *STR*, *TDC* and *SLTr* (Supplementary Table 6). **b**, Chemical scheme showing tryptamine, secologanin, strictosidine and secologanin and VIGS for *SLTr*. Bar heights represent means; error bars represent s.e.m. Each dot represents a sample. *P* values are based on two-tail Tukey tests and are shown on the graphs. EV, empty vector control. EV, *n* = 8. VIGS, *n* = 6. **c**, Hi-C contact map for a gene cluster consisting of *GS1*, *GS2*, *THAS2* and *PAS*. The curve represents the chromosome loop. **d**, Hi-C contact map for a gene cluster consisting of an array of acetyltransferases. 1, 2 and 3 represent three TADs. Three previously studied biosynthetic genes (*MAT*, *TAT* and *DAT*) are indicated by asterisks. **e**, Gene expression profiles for acetyltransferases highlighted in **c**. FPKM: fragments per kilobase exon mapped per million reads. Heatmap represents Hi-C contacts at the 10-kb resolution, where the darker color represents higher Hi-C contacts (**a**,**c**,**d**). Color scales are maxed out at 100 Hi-C contacts per 10 kb. Solid lines and *P* values represent TAD boundaries detected by HiCKey^12^. *P* values are derived from generalized likelihood ratio tests, part of the HiCKey workflow, and are shown on the graphs. See Supplementary Fig. 1 and Supplementary Table 6 for abbreviation definitions. Source data

The highly contiguous v3 assembly revealed clusters of MIA pathway genes (Supplementary Tables 6 and 7). Two clusters were identified, a paralog array containing tetrahydroalstonine synthase 1 (*THAS1)*, *THAS3*, *THAS4* and the *THAS* homolog, heteroyohimbine synthase, as well as a cluster containing serpentine synthase (*SS*)^9^, an *SS* paralog with near identical protein sequence, strictosidine glucosidase (*SGD*) and *SGD2*. Gene duplications are major drivers of chemical diversity^10^, and a total of 207 paralogs of 69 genes previously implicated in MIA biosynthesis were identified in the v3 genome, of which, a substantial number were locally duplicated (Supplementary Table 7). Interestingly, paralogs of MIA biosynthetic genes were identified within the *SS–SGD–SGD2* cluster, the tabersonine 16-hydroxylase (*T16H2*)*-*16-hydroxytabersonine O-methyltransferase (*16OMT*) cluster and the strictosidine synthase *(STR*)*-*tryptophan decarboxylase (*TDC*) gene clusters. Notably, a multidrug and toxic compound efflux transporter (*MATE*) was also located in the *STR–TDC* cluster (see also Fig. 3a,b). In summary, the high-quality *C. roseus* v3 genome assembly and annotation provide the foundation for accelerated discovery of the final MIA biosynthetic pathway genes and the mechanisms underlying the complex organ and cell-type-specific gene regulation of this pathway.

### Gene clusters and chromosome conformation features

Unlike biosynthetic gene clusters found in prokaryotic genomes, biosynthetic gene clusters in plant genomes have a loose organization, with unrelated genes or long intergenic spaces separating the biosynthetic genes. Nevertheless, these gene clusters both facilitate gene identification and are believed to have a role in transcriptional regulation^11^. With the recent advent of mapping chromosome conformation features, we now have the ability to probe the location of genes in 3D space. Thus, in addition to searching for biosynthetic gene clusters linearly organized on the chromosome, we can also search for biosynthetic genes that are confined within the same 3D space.

Using the v3 *C. roseus* genome assembly, we probed chromatin interactions between biosynthetic genes in 3D space using Hi-C data from mature leaves. HiCKey^12^ was used to detect topologically associated domains (TADs) and HiCCUPS to detect chromosome loops^13^, revealing distinct chromosomal organizations associated with biosynthetic gene clusters. For example, *STR* and *TDC* are physically clustered on chromosome 3 (Fig. 3a). STR and TDC are consecutive steps along the pathway, where TDC catalyzes the formation of tryptamine from tryptophan and STR catalyzes the condensation between secologanin and tryptamine to form strictosidine. The *MATE* transporter that is located adjacent to *TDC* in the linear genome was also colocated within a TAD with *STR* and *TDC*, as indicated by a high level of long-distance contacts detected by Hi-C (Fig. 3a). To test whether this transporter is involved in MIA transport, we performed virus-induced gene silencing (VIGS) of this gene in young *C. roseus* leaves (Supplementary Fig. 4a). Although the levels of secologanin and downstream alkaloids did not change substantially in response to silencing, we detected a build-up of a compound with *m/z* 391 (M + H)^+^ and *m/z* 413 (M + Na)^+^ in the *MATE-*silenced tissue but not in empty vector controls (Fig. 3b; *P* = 1.3 × 10^−9^, Tukey’s tests). This compound was assigned as secologanol based on co-elution with a standard obtained by chemical reduction of secologanin (Supplementary Fig. 4b). The most likely explanation of the VIGS chemotype is that this *MATE* transporter transports secologanin from the cytosol into the vacuole, where *STR* is localized. The lack of secologanin transport would result in a build-up of secologanin in the cytosol, where the reactive aldehyde would be reduced to the less toxic secologanol. Thus, we named this *MATE* transporter *SLTr*. Unfortunately, all our attempts to heterologously express *SLTr* for in vitro transport assays were unsuccessful. This gene cluster has been observed in other MIA producers *Gelsemium sempervirens* and *Rhazya stricta*, suggesting that it is conserved across strictosidine-producing plants^7^. We also observed that biosynthetic genes interact in 3D space via chromosome loops as shown with *THAS2* and precondylocarpine acetate synthase (*PAS*)^14,15^, which are separated by ~50 kb in the linear distance (Fig. 3c).

Not all genes in a biosynthetic gene cluster are in the same TAD. For example, an array of locally duplicated acetyltransferases was found on chromosome 2, including three that were previously characterized (minovincinine-19-hydroxy-O-acetyltransferase (*MAT*), tabersonine derivative 19-O-acetyltransferase (*TAT*) and deacetylvindoline O-acetyltransferase (*DAT*)). This array of acetyltransferases is separated into three TADs, with *MAT* and *TAT* within TAD 2 and *DAT* within TAD 3 (Fig. 3d)^16,17^. This segregation of acetyltransferases within TADs coincides with organ-level expression patterns; *MAT* and *TAT* are expressed in roots but not in leaves, and *DAT* is expressed in leaves but not in roots (Fig. 3e). These observations suggest chromosome conformation may have regulatory roles in controlling organ-specific biosynthetic gene expression, and consequently, the localization pattern of specialized metabolite production.

### Gene expression at cell type resolution in leaves

**Fig. 4:**
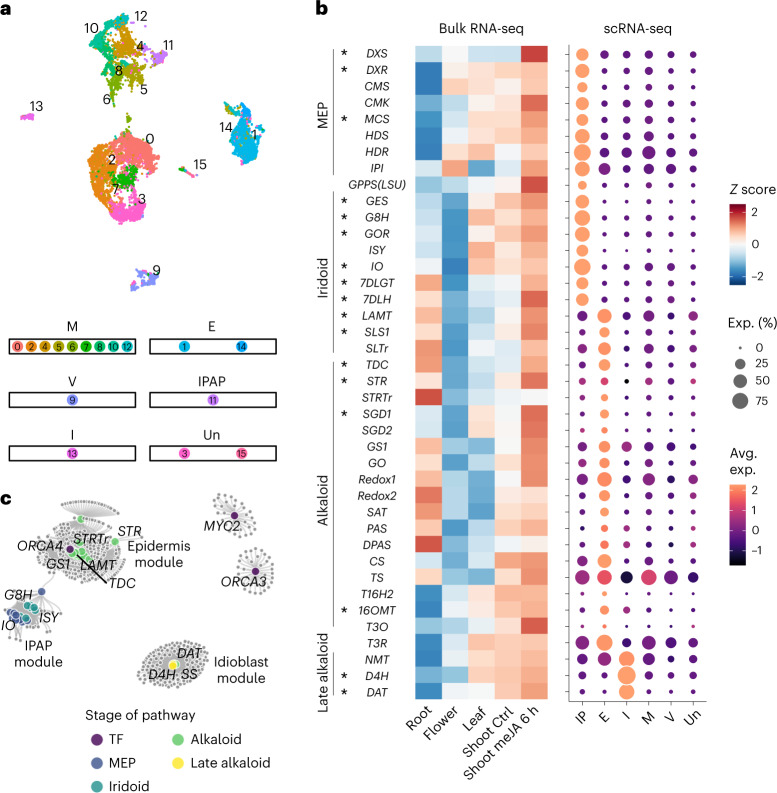
MIA biosynthetic genes are partitioned into three discrete cell types in *the C. roseu*s leaf. **a**, UMAP of gene expression in *C. roseus* leaves (*n* = 15,437 cells). **b**, Gene expression heatmap of the MIA biosynthetic pathway for bulk and single-cell transcriptomes. Genes are arranged from upstream to downstream. Previously reported cell-type-specific expression^18–22^ are confirmed and marked with asterisks. For the single-cell gene expression heatmap, color scale shows the average scaled expression of each gene at each cell type (Methods). Dot sizes indicate the fraction of cells where a given gene is expressed at a given cell type. **c**, Gene coexpression network for MIA biosynthetic genes using leaf scRNA-seq data. Each node is a gene. Larger size nodes represent previously characterized genes. Edges represent coexpression (FDR < 0.01; Methods). See Supplementary Fig. 1 and Supplementary Table 6 for a list of gene name abbreviations. There are 38 biosynthetic genes and two transporters, among which SLTr and STRTr are transporters. See Supplementary Table 9 for the membership of genes in each module. E, epidermis; I, idioblast; M, mesophyll; V, vasculature; Un, unassigned. Source data

In situ hybridization experiments have established the expression specificity of a subset of the 38 known biosynthetic genes involved in bis-indole alkaloid biosynthesis, where the initial steps are located in internal phloem-associated parenchyma (IPAP) cells, downstream enzymes in epidermis and late enzymes located in idioblast cells^18–22^. We performed single-cell RNA-sequencing (scRNA-seq) on ~13 to 14-week-old *C. roseus* leaves (Supplementary Fig. 5) and obtained gene expression profiles of 15,437 cells and 19,337 genes (Fig. 4a). We integrated three independent biological replicates using Seurat^23^ (Supplementary Fig. 6) with clustering patterns similar across three replicates. Cell types were assigned using Arabidopsis marker gene orthologs (Supplementary Table 8, Fig. 4a and Supplementary Fig. 6). Two cell types, IPAP and idioblasts, were inferred using previously studied *C. roseus* biosynthetic genes that show cell-type-specific expression^18,19^. In an independent experiment, we profiled gene expression at the single-cell level across 1,379 cells using the Drop-seq platform^24^ (Supplementary Fig. 7a). Although fewer cell types were detected using the Drop-seq platform, expression profiles across the top 3,000 variable genes were highly concordant between the cell types detected by two different platforms (Supplementary Fig. 7b). Taken together, we inferred that the single-cell expression profiles are robust and reproducible across two experimental platforms.

Coexpression analyses using whole-tissue or organ-derived gene expression datasets, that is, bulk mRNA-seq, have enabled the discovery of MIA biosynthetic pathway genes (Fig. 4b). However, whole organ/tissue-derived expression abundances provide an expression estimation that is averaged over all cells in the organ. Thus, if a gene is expressed in a rare cell type, such as the idioblast, the power of coexpression analyses will be limited at best. Therefore, we examined scRNA-seq data for biosynthetic gene expression across cell types and found that the pathway is clearly expressed in three specific leaf cell types (Fig. 4b). The improved resolution in cell-type-specific expression profiles compared to tissue-specific profiles is stark (Fig. 4b and Supplementary Fig. 7c). The cell-type-specific expression patterns of 17 biosynthetic genes previously characterized by mRNA in situ hybridization^18–22^ (Fig. 4b, marked with *) confirmed our cell type resolution of MIA biosynthesis. The MEP and iridoid stages of the pathway are expressed in IPAP, whereas the alkaloid segment of the pathway is primarily expressed in the epidermis, and the final known steps of the pathway are exclusively expressed in the idioblasts. Previous work suggested that a heterodimeric GPPS, composed of large subunit (LSU) and small subunit (SSU), is responsible for geranyl pyrophosphate used in MIA biosynthesis^25^. Interestingly, although GPPS LSU is specifically expressed in IPAP, GPPS SSU is expressed in other cell types as well (Supplementary Fig. 7d). Finally, we found that secologanin transporter (*SLTr)* that is physically clustered with *TDC* and *STR* and colocated within the same TAD as these two biosynthetic pathway genes are specifically expressed in the epidermis (Fig. 3a,b and Fig. 4b), further supporting its involvement in transporting the MIA intermediate secologanin.

We performed gene coexpression analyses producing a network graph for biosynthetic genes as well as previously reported transcription factors. The network is self-organized into three main modules, corresponding to IPAP, epidermis and idioblast (Fig. 4c). We also note that *SS*^9^ is a member of the idioblast coexpression module. SS catalyzes the formation of serpentine, which has a strong blue autofluorescence and has been previously used as a visual marker for idioblast^9^. The recovery of SS in the idioblast module confirms the robustness of this coexpression analysis. Upon jasmonic acid (JA) elicitation, *MYC2* and *ORCA3* transcription factors are activated, which in turn activate MIA biosynthetic genes^26^. However, *MYC2* and *ORCA3* were not part of any modules containing biosynthetic genes (Fig. 4c), suggesting that the regulatory mechanisms in response to JA are distinct from those controlling cell-type-specific expression. Finally, a paralog of *ORCA3*, *ORCA4* (ref. ^27^), was detected within the epidermis module, suggestive of regulatory roles beyond JA-responsiveness. Gene identifiers of all genes within coexpression modules shown in Fig. 4c can be found in Supplementary Table 9. Taken together, the leaf scRNA-seq dataset is consistent with data obtained from previously established localization methods and provides accurate and high-resolution data for gene discovery. Moreover, the cell type resolution expression patterns produced coexpression networks that clarified and expanded regulatory relationships.

### High-throughput scMet

Single-cell mass spectrometry (scMS) has lagged behind scRNA-seq due to the intrinsic limitations related to the abundance of the analytes, which is exacerbated by the fact that metabolites cannot be amplified like RNA or DNA. Because the volume of single cells is low (fL to nL range), even when intracellular analytes are present at millimolar concentrations, mass spectrometry detection methods require extreme sensitivity. Although progress has been made in the development of scMS approaches^28^, few methods have been successfully applied to plant cells. To date, mass spectrometry analyses of individual plant cells have either relied on MS imaging, which is hindered by low spatial resolution, complex sample preparation protocols and low throughput, or live single-cell mass spectrometry (LSC-MS) method^29,30^, which is highly labor-intensive and not high throughput. None of these methods uses chromatographic separation before mass spectrometry analysis, greatly limiting accurate structural assignment and quantification of metabolites.

**Fig. 5:**
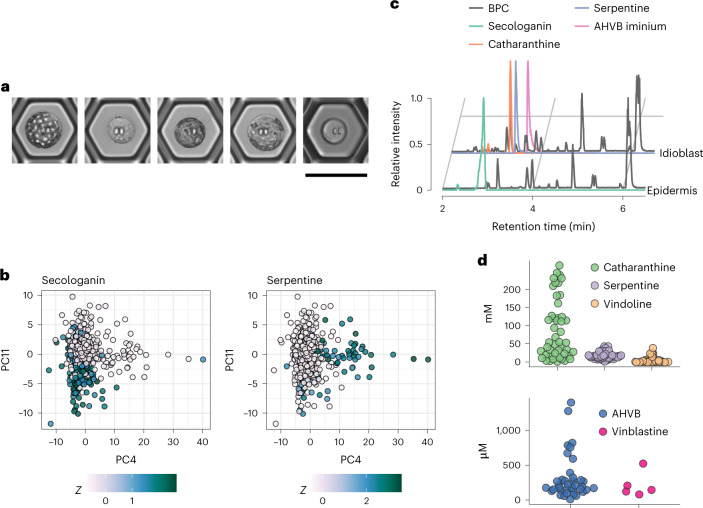
Single-cell metabolomic analysis of leaves. **a**, Photos of some isolated protoplasts. Scale bar = 50 µm. **b**, Principal component plots colored by *Z* scores of secologanin and serpentine (*n* = 586 cells). **c**, UPLC/–MS traces of selected metabolites for idioblast and epidermal cells. BPC of the representative epidermis and idioblast cells. BPC, base peak chromatogram. **d**, Concentration estimates of selected compounds in single cells where they were detected. Each dot is a single cell. Catharanthine, *n* = 47; vindoline, *n* = 47; serpentine, *n* = 47; AHVB, *n* = 37 and vinblastine, *n* = 5. Source data

To address these limitations, we designed a process in which a high-precision microfluidic cell-picking robot was used to collect protoplasts prepared from *C. roseus* leaves from a Sievewell device (Supplementary Fig. 8). Protoplasts were then transferred to 96-well plates compatible with an ultra-high liquid chromatography–mass spectrometry (UPLC–MS) autosampler. The UPLC–MS method was optimized using available MIA standards (Supplementary Table 10). For this study, we collected a total of 672 single cells in seven 96-well plates that were each subjected to UPLC–MS, allowing simultaneous untargeted and targeted metabolomic analysis. As the analysis of all cells was performed over several days, we treated each 96-well plate as an independent experiment, to control for batch-to-batch variation due to experimental and instrumental variables. After close inspection of the selected cells, 86 samples were removed as they either contained two cells, none or some debris, reducing the total number of cells included in the analysis to 586. Representative examples of different cells collected in the experiment are shown in Fig. 5a.

When using an intensity threshold of 5 × 10^4^ counts, 34,729 peaks were detected by XCMS software package. We used CAMERA to group redundant signals (isotopes, adducts, etc.) and kept only the most widely detected peaks, yielding 8,268 representative features. Finally, we excluded the peaks that were not detected in all batches, for a total of 933 features. Raw areas were corrected by the intensity of the internal standard (ajmaline), log-transformed and center-scaled by batch to minimize batch-to-batch artifacts. Principal component analysis (PCA) unambiguously separated a group of cells that could be assigned as idioblast cells based on the occurrence of serpentine (*m/z* 349.1547 (M)^+^, C_21_H_20_N_2_O_3_) and vindoline (*m/z* 457.2333 (M + H)^+^, C_25_H_32_N_2_O_6_), which have been previously reported to localize to idioblast cells^29–31^ (Fig. 5b). Another group of cells, the epidermal cells, could be identified based on the occurrence of the secoiridoid secologanin (*m/z* 389.1442 (M + H)^+^, C_17_H_24_O_10_), known to be synthesized in epidermal cells^32^ (Fig. 5b). Strictosidine, which is formed from secologanin and also synthesized in the epidermis, was observed at low levels in only a few cells, because this compound accumulates in leaves younger than those used here. No iridoid intermediates were detected under these conditions, but iridoid intermediates do not accumulate at substantial levels and do not ionize efficiently in ESI^+^. Representative chromatograms of an idioblast and of an epidermal cell are shown in Fig. 5c and Supplementary Fig. 9. MS/MS fragmentation experiments, conducted on pooled cells quality control (QC) samples, allowed unambiguous identification of key MIAs, such as catharanthine (*m/z* 337.1911 (M + H)^+^, C_21_H_24_N_2_O_2_), vindorosine (*m/z* 427.2227 (M + H)^+^, C_24_H_30_N_2_O_5_), vindoline and serpentine (Supplementary Fig. 10). Comparison between the methanolic extract of the leaf tissue used to generate the protoplasts, and the protoplasts used for cell picking showed that the metabolite profile is not altered during the process of protoplast preparation (Supplementary Fig. 11).

External calibration using authentic standards allowed the quantification of selected metabolites within the cells (Supplementary Table 11). The concentrations of the analytes were corrected for the volume of the cells, calculated from the cell dimensions that were measured during the picking process. Surprisingly, the concentration of the major metabolites accumulating in idioblasts was in the millimolar range. Although large differences in concentration were observed in the individual cells, the average catharanthine concentration was 100 mM (Fig. 5d), which is unexpected because the enzyme that produces this metabolite (catharanthine synthase (CS)) is located in the epidermis. We detected only low amounts of catharanthine in a few epidermal cells, and live cell mass spectrometry discussed in refs. ^29,30^ also demonstrated that the majority of this MIA accumulates in the idioblast cells. Catharanthine was proposed to be exported from the epidermal cells to the cuticle through the action of the ABC transporter CrTPT2 (ref. ^33^), but although our mass spectrometry method could not measure metabolites on the leaf cuticle, our data clearly show that substantial amounts of catharanthine are sequestered inside leaf idioblast cells. Thus, we hypothesize that catharanthine is rapidly transported from the epidermis to the idioblasts. In contrast, the location of most detected MIAs, including vindoline, vindorosine and serpentine, corresponded perfectly with the cell type expression of their biosynthetic enzymes^9,34^. Intracellular quantification of metabolite levels will allow a better understanding of the enzyme kinetic properties in vivo and of the rates of metabolic reactions, although subcellular compartmentalization and transport will also have to be taken into account. For instance, some metabolites, such as secologanin and vindoline, are synthesized in the cytoplasm and stored in the vacuole, making the correlation of concentration with steady-state enzyme kinetic parameters difficult. Nevertheless, we believe this methodology has great potential to determine the extent of substrate saturation of metabolic enzymes and to study the free energy of metabolic reactions.

Our analysis also targeted anhydrovinblastine, vinblastine and vincristine (bis-indole alkaloids), which had also been detected in idioblast cells using LSC-MS^29^. Anhydrovinblastine (*m/z* 397.2122 (M + 2H)^2+^, C_46_H_56_N_4_O_8,_ AHVB) was detected in the micromolar range in almost all of the idioblast cells analyzed, and its MS/MS fragmentation confirmed its identity (Supplementary Fig. 12). However, vinblastine (*m/z* 406.2175 (M + 2H)^2+^, C_46_H_58_N_4_O_9_) was found only in five cells, and vincristine was not detected at all (Supplementary Fig. 13). This reflects the low levels in which vinblastine and vincristine accumulate. However, catharanthine and vindoline, the proposed precursors of the bis-indole alkaloids, co-occur in the same cell type in which AHVB and vinblastine are present, suggesting that the enzymes involved in bis-indole biosynthesis should also be present in the idioblasts. Because concentrations of catharanthine and vindoline are two orders of magnitude higher than AHVB and vinblastine, it is likely that the coupling reaction leading to the bis-indole alkaloids is a rate-limiting step, which could be due to low expression, low specific activity of the coupling enzyme, or that coupling is hindered by intracellular compartmentalization of the two monomers.

**Fig. 6:**
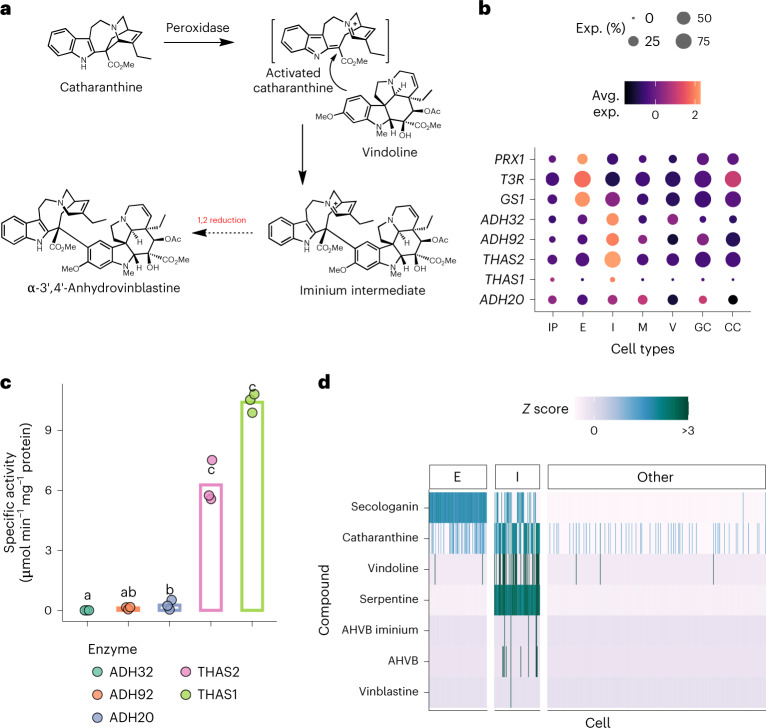
Reduction of an iminium to form anhydrovinblastine. Discovery of ADH20, and comparison of kinetic parameters against THAS2 and other ADHs. **a**, A short chemical scheme showing coupling and reduction. **b**, Expression heatmap at single-cell type resolution. Color scale shows the average scaled expression of each gene at each cell type (Methods). Dot sizes indicate the fraction of cells where a given gene is expressed at a given cell type. **c**, Specific activity of the five idioblast-localized ADH enzymes recombinantly expressed in *E. coli* and tested in vitro for activity toward the AHVB iminium. *n* = 3 for all assayed enzymes. **d**, Heatmap showing cells as columns and compounds as rows from the scMet experiment (*n* = 586 cells). Source data

The fact that AHVB and vinblastine levels do not correlate with upstream pathway intermediates is likely a major reason why the late-stage enzymes that convert vindoline and catharanthine to AHVB and vinblastine have been particularly challenging to elucidate (Fig. 6a). In this coupling reaction, an oxidase activates catharanthine, which then reacts with vindoline to form an iminium dimer. A reductase is then required to reduce this iminium species to form AHVB. Our scMet analysis revealed the presence of a chemical species with *m/z* 396.2044 (M^+^ + H)^2+^ and chemical formula C_46_H_54_N_4_O_8_^+^, consistent with this iminium dimer in idioblast cells, along with the monomers and AHVB (Supplementary Fig. 14). The mechanism of enzymatic reduction of the iminium intermediate to AHVB is not known, but recent work in our group has identified a number of medium-chain alcohol dehydrogenases in *C. roseus* that can reduce iminium moieties (GS, Redox1 and T3R; Supplementary Figs. 1 and 2). Therefore, we hypothesized that this reduction of the iminium dimer would be carried out by a medium-chain alcohol dehydrogenase localized to idioblast cells. From the coexpression modules across cell types (Fig. 4c), we identified five idioblast-specific medium-chain alcohol dehydrogenases (Fig. 6b). These enzymes were heterologously expressed in *Escherichia coli* and assayed with the iminium dimer, which could be generated in vitro by incubating catharanthine and vindoline with commercial horseradish peroxidase, which is known to catalyze the initial oxidative coupling^35^. Two enzymes, THAS1 and THAS2, formed AHVB when incubated with the iminium dimer, with specific activities of 10.38 ± 0.27 μmol min^−1^ mg^−1^ and 6.26 ± 0.62 μmol min^−1^ mg^−1^, respectively (Fig. 6c). We attempted to demonstrate the function of THAS1 and THAS2 in planta by silencing them using VIGS. However, silencing of *THAS1* or *THAS2* individually or simultaneously did not substantially affect the levels of vinblastine, AHVB and the iminium dimer (Supplementary Figs. 15 and 16). Therefore, although the in vitro function of these enzymes is clear, we cannot definitively define a physiological function for them. It is important to note however, that a number of medium-chain alcohol dehydrogenases from the MIA pathway (GS and T3R) have also failed to show strong chemical phenotypes when subjected to VIGS ^36,37^. Both THAS1 and THAS2 have been previously biochemically characterized as tetrahydroalstonine synthases, an enzyme that generates tetrahydroalstonine from the reduction of strictosidine aglycone. Notably, however, tetrahydroalstonine levels in the leaf are low, which is consistent with an alternative catalytic function for these enzymes in the leaf.

A peroxidase, CrPRX1, that can activate catharanthine to form the iminium intermediate, has been previously reported in ref. ^38^. Surprisingly, this enzyme is selectively expressed in the epidermis (Fig. 6b), in contrast to the localization of vindoline, iminium dimer and AHVB (Fig. 6d). Notably, the dimerization reaction can be catalyzed by nonspecific peroxidases, such as horseradish peroxidase, so we hypothesize that CrPRX1 is also a nonselective enzyme that has another function in planta. We did not identify any idioblast-specific peroxidase in the leaf scRNA-seq dataset. These omics datasets set the stage for future work, which will focus on functionally characterizing additional classes of oxidases—which are challenging to functionally express in heterologous systems—specifically localized to the idioblast for activity in coupling and oxidation to vinblastine.

### Root single-cell transcriptome

**Extended Data Fig. 1:**
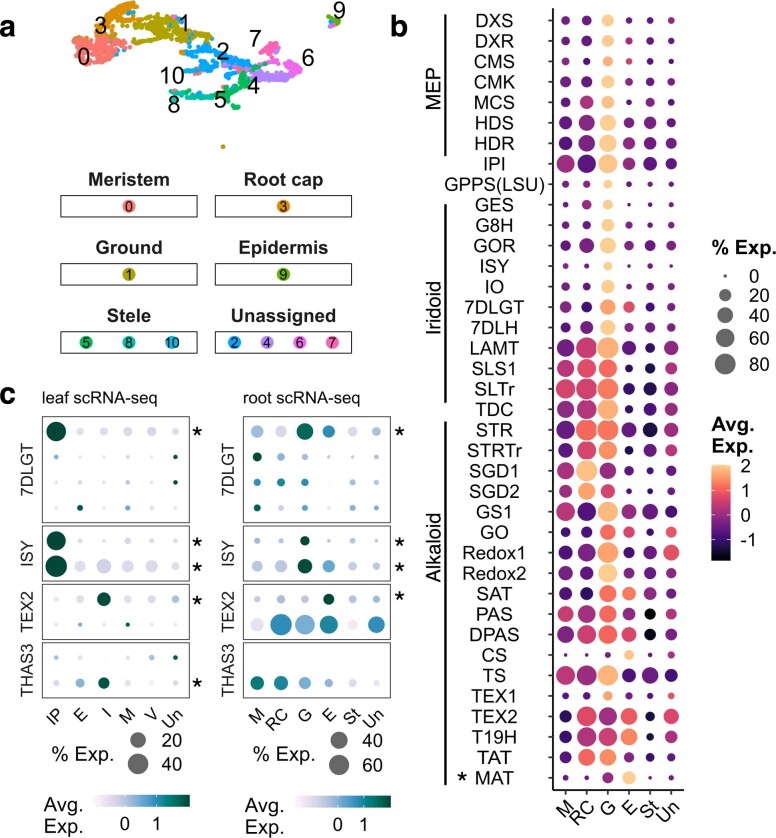
Single cell transcriptome of *C. roseus* roots highlights differential regulation between organs. **a**, UMAP (uniform manifold approximation and projection) for *C. roseus* roots (*n* = 2636 cells). **b**, MIA biosynthetic gene expression heatmap: Genes are arranged from upstream to downstream. Previously reported cell-type-specific expression in roots^17^ is marked with asterisks. **c**, Comparison of cell-type-specific expression pattern between leaf and root among tandemly duplicated paralogs. Each row is a paralog, organized by gene family. Columns are cell types, grouped by organ. Asterisks represent the paralog that is expressed along with the rest of the pathway. Color scales show the average scaled expression of each gene at each cell type (see also Methods). Dot sizes indicates the fraction of cells where a given gene is expressed at a given cell type. See Supplementary Table 6 for a list of gene abbreviations. Ground tissue includes cortex and endodermis cell types. M: meristem; RC: root cap; G: ground tissue; E: epidermis; St: stele; Un: unassigned. Source data

We also performed scRNA-seq on *C. roseus* roots to compare cell-specific expression in two distinct organs. Although catharanthine and tabersonine are present in both organs, the derivatization of tabersonine diverges in root and leaf (Supplementary Figs. 1 and 2). The tabersonine-derived product vindoline, which goes on to form AHVB, is found in leaves, while tabersonine-derived hörhammercine is found in roots (Supplementary Figs. 1 and 2). The root scRNA-seq dataset captured the expression of 2,636 cells and 18,190 genes from two biological replicates that grouped into 11 clusters and six major tissue classes (Extended Data Fig. 1a). The clustering patterns are highly similar between the two replicates (Supplementary Fig. 17a). *MAT* was previously reported by in situ hybridization^17^ to be expressed in epidermis and cortex. In the root scRNA-seq data, *MAT* was also found to have dual localization (cluster 4 and cluster 8, and Supplementary Fig. 17b). Cluster 4 contained marker genes for both endodermis and cortex (*PBL15* and *AED3*) ^39^, whereas cluster 8 contained maker genes for atrichoblast epidermis (*TTG2* and *GL2*)^3,39,40^ (Supplementary Fig. 17c and Supplementary Table 8). Collectively, these results recapitulate a dual-expressed biosynthetic gene previously characterized by in situ hybridization.

The spatial organization of the core *MIA* genes differed between leaf and root, highlighting the plasticity of cell-specific regulation in these two organs. In leaves, the MIA pathway switched from IPAP to epidermal cells at loganic acid methyltransferase (*LAMT*) and from epidermal to idioblast cells at 3-hydroxy-16-methoxy-2,3-dihydrotabersonine N-methyltransferase (*NMT*) (Fig. 3b), but in roots, the pathway was not partitioned into three discrete cell types (Extended Data Fig. 1b). Instead, the MEP and iridoid stages are specifically expressed in the ground tissue composed of cortex and endodermis (Extended Data Fig. 1b and Supplementary Fig. 18a) with an expression of the alkaloid stage, while also expressed in ground tissues, exhibiting a more diffused expression pattern (Extended Data Fig. 1b and Supplementary Fig. 18a). Parallel to vindoline biosynthesis in leaves, the late-stage derivatization enzymes that modify tabersonine to hörhammercine are found in a different cell type from the rest of the pathway genes. Tabersonine 6,7-epoxidase *(TEX*)^16^, tabersonine 19-hydroxylase^41^ and *TAT*^42^ all have a detectable expression in the epidermis, along with *MAT*^17^ (Supplementary Fig. 18a). Our root scRNA-seq experiment used highly developed 8-week-old whole root systems that are much more complex than root tips of Arabidopsis seedlings from which the root marker genes for dicots are derived. For example, secondary growth has not occurred in Arabidopsis seedling root tips, while in 8-week-old *C. roseus* roots, we clearly observe secondary growth, which may explain the presence of unassigned cell types in our root dataset (Extended Data Fig. 1a). Despite the highly developed state of the root tissue, we nonetheless observed cell-type-specific expression of the MIA pathway primarily in the ground tissue, with the final reaction(s) present in the root epidermis (Extended Data Fig. 1b and Supplementary Fig. 18a).

The v3 assembly resolved multiple tandemly duplicated paralogs, some of which were collapsed or fused in the v2 assembly. To clarify the potential functions of these paralogs, we compared their expression patterns in both leaf and root across cell types. We noticed examples in which a single paralog was preferentially expressed in a given cell type compared to the other paralog(s). For example, a single, collapsed *7DLGT* in v2 was resolved into four separate loci in v3 that displayed cell-type-specific expression patterns in leaf and root revealing neo- or subfunctionalization at the expression level (Extended Data Fig. 1c and Supplementary Fig. 18b). We also observed retention of expression patterns among paralogs. Iridoid synthase (*ISY*), well characterized by silencing in the leaf, has a tandemly duplicated paralog. Although the *ISY* paralog is expressed in leaf tissue, silencing showed no obvious changes^43^, which can be explained by the redundant expression of both paralogs. In the root, both paralogs are expressed in the ground tissue along with the rest of the pathway. However, one of the paralogs is expressed in a higher percentage of cells (Extended Data Fig. 1c). Finally, both *TEX2* and *THAS3* (ref. ^14^) have a tandemly duplicated paralog for which cell type resolution expression patterns highlighted which paralog is likely involved in the biosynthesis of MIAs (marked by asterisk in Extended Data Fig. 1c).

## Discussion

Over the last 15 years, next-generation sequencing technologies allowed the rapid generation of transcriptomic and genomic datasets that facilitated gene discovery in many plant species. Here we report how state-of-the-art omics methods not only accelerate gene discovery through the high-resolution spatial resolution of gene expression but also how complementary omics data facilitates the construction of a more holistic view of the genome encapsulating genes, gene regulation in 2D (linear) and 3D (chromatin) space, and genic end products, that is, metabolites.

Generation of a highly contiguous, chromosome-scale genome assembly revealed substantial duplication of MIA biosynthetic pathway genes, of which, some are clustered in the linear genome. Chromatin interaction maps revealed 3D clustering of a subset of MIA pathway genes, some exhibiting organ-level specificity and interactions via chromatin loops, suggesting that TADs, in addition to physical colocalization, can be used to identify genes involved in specialized metabolism. The detection of TADs and chromatin loops of physically clustered genes is consistent with the coregulation hypothesis on the origins of biosynthetic pathway gene clustering as the 3D chromatin interactions serve key roles in gene regulation^44^. The ability to detect cell-type-specific gene expression data revealed that the MIA pathway is spatially and sequentially partitioned across discrete cell types in *C. roseus* leaf, permitting the construction of cell-type-specific coexpression modules for IPAP, epidermis and idioblast cells. Notably, the cell type expression profile of MIA biosynthesis genes in leaf and root are different, highlighting the plasticity of the gene expression networks between organs as well as the neo- and subfunctionalization at the gene expression level of MIA biosynthetic pathway paralogs.

Aside from the MIA, multicellular localization patterns of only a few specialized metabolite pathways have been investigated in full. In the morphine biosynthetic pathway, biosynthetic enzymes are synthesized in companion cells, which are then delivered to sieve elements, where the early steps of the pathway take place. Later-stage intermediates are then transported from the sieve elements to laticifers where the enzymes involved in the late steps of the morphine pathway are localized, which is also the site of morphine and other alkaloid accumulation^45^. Additionally, the localization of the glucosinolate pathway in *Arabidopsis thaliana* has been established^4^. Biosynthetic enzymes for aliphatic glucosinolate biosynthesis appear to be located in xylem parenchyma cells and phloem cells, while indole glucosinolate biosynthetic enzymes are localized to sieve element-neighboring parenchyma cells of the phloem, and glucosinolates are transported to and stored in S-cells.

The reasons for the distinct localization of the MIA pathway are not known. The spatial organization of natural products could have an important role in how these metabolites function in defense or signaling. Notably, strictosidine and strictosidine glucosidase, which likely serve an antifeedant role^46^, are located in the epidermis. More derivatized alkaloids, which do not yet have a known ecological role, appear to be derivatized and then stored in the idioblasts, comparable to the role laticifers have in benzylisoquinoline alkaloid biosynthesis. Alternatively, the localization pattern may simply be an accident of evolution, in which the biosynthetic enzymes have evolved from pre-existing enzymes located in these cell types. The partitioning that is observed in MIA biosynthesis does not appear to serve any obvious chemical function, such as separating intermediates that may cross-react.

The high-throughput mass spectrometry method developed here showed not only which metabolites co-occurred in distinct cell types but also allowed us to measure the concentrations of metabolites across a cell population. Notably, although catharanthine is synthesized in the epidermis (as evidenced by the localization of the biosynthetic enzyme CS), this alkaloid colocalizes with vindoline, which is synthesized in idioblasts (as evidenced by the localization of the biosynthetic enzymes D4H and DAT). Therefore, catharanthine must be intercellularly transported from the epidermis to the idioblast. Notably, the concentration of catharanthine and vindoline, which dimerize to form AHVB, were in the high millimolar range. In contrast, the dimerization product AHVB was in the micromolar range, indicating that the coupling step is rate-limiting. This discovery serves as a starting point to design strategies to genetically engineer *C. roseus* plants with higher levels of AHVB.

These state-of-the-art omics datasets provide a foundation for the discovery of the remaining genes and regulatory sequences involved in cell-type-specific MIA biosynthesis and transport in *C. roseus*. One-quarter of all pharmaceuticals are derived from plants^47^; here we show the power of single-cell multi-omics in natural product gene discovery in plants. We anticipate that application of complementary single-cell omics methods will be essential in tapping the wealth of chemistry present across the plant kingdom.

## Methods

### Genome sequencing and assembly

*C. roseus* cv ‘Sunstorm Apricot’ was grown under a 15 h photoperiod at 22 °C and dark-treated for 36 h before harvesting leaves from 17-week-old plants. High-molecular-weight DNA was isolated using a QIAGEN Genomic-tip 500/G after a crude cethyltrimethyl ammonium bromide extraction^48^. ONT libraries were prepared using the ONT SQK-LSK110 kit and sequenced on R9 FLO-MIN106 Rev D flow cells; the latest software available at the run date for each library was used (Supplementary Table 4). ONT whole-genome shotgun libraries were base-called using Guppy (5.0.7 + 2332e8d, https://nanoporetech.com/community) using the high-accuracy model (dna_r9.4.1_450bps_hac). Reads less than 10 kb were filtered out using seqtk (v1.3, https://github.com/lh3/seqtk), and remaining reads greater than 10 kb were assembled with Flye (v2.8.3-b1695)^49^ using the parameters -i 0 and --nano-raw. The assembly was polished with two rounds of Racon (v1.4.20)^50^, followed by two rounds of Medaka (v1.4.3, https://github.com/nanoporetech/medaka) using the ‘r941_min_hac_g507’ model, and finally, three rounds of Pilon (v1.23)^51^ using Illumina whole-genome shotgun reads (Supplementary Table 4).

Hi-C libraries were constructed from immature leaf tissue grown under a 15 h photoperiod with constant 22 °C conditions following manufacturer recommendations using the Arima Hi-C 2.0 Kit (Arima Genomics; CRO_AN, CRO_AO; Supplementary Table 4). Hi-C libraries were sequenced on an S4 flow cell in paired-end mode generating 151 nucleotides (nt) reads on an Illumina NovaSeq 6000 (Illumina). Contigs less than 10 kb were removed from the assembly using seqtk (v1.3; https://github.com/lh3/seqtk). Pseudochromosomes were constructed using the Juicer (v1.6)^52^ and 3D-DNA (git commit 429ccf4)^53^ pipelines using the Illumina Hi-C sequencing data with default parameters.

To produce the target file for adaptive finishing, 5-kb ends of contigs from the primary assembly were used (full sequences for contigs <10 kb in size) in the first run, while in the second run, 30-kb ends from contigs were used (full sequences for contigs <60 kb in size). In the second run, half of the channels in the flow cell were set to adaptively sample. Base-calling was performed with Guppy v5.0.16 (nanoporetech.com/community) with the following parameters: --config dna_r9.4.1_450bps_hac.cfg --trim_strategy dna --calib_detect. seqtk v1.3 (github.com/lh3/seqtk) was used to filter reads that were adaptively sampled (not rejected) by the pore. Adaptive finishing reads and the bulk ONT genomic reads were used to fill in gaps in the pseudomolecules and unanchored scaffolds using the DENTIST pipeline (v3.0.0)^54^ with the following parameters: read-coverage: 90.0, ploidy: 2, max-coverage-self: 3 and join-policy: scaffoldGaps.

### Genome annotation

A custom repeat library was created using RepeatModeler (v2.0.3)^55^. Putative protein-coding genes were removed using ProtExcluder (v1.2)^56^, and Viridiplantae repeats from RepBase^57^ v20150807 were added to create the final custom repeat library. The final genome assembly was hard-masked and soft-masked using the custom repeat library and RepeatMasker (v4.1.2)^58^.

To provide transcript evidence for genome annotation and gene expression abundance estimations, publicly available mRNA-seq libraries were downloaded from National Center for Biotechnology Information (Supplementary Table 4). RNA-seq libraries were processed with Cutadapt (v2.10)^59^ with the following parameters: --minimum-length 75 and –quality-cutoff 10. Cleaned reads were aligned to the assembly with HISAT2 (v2.1.0)^60^ with the following parameters: --max-intronlen 5000 --rna-strandness ‘RF’ --no-unal --dta; transcript assemblies were generated from the alignments using StringTie2 (v2.2.1)^61^. FL-cDNA sequences were generated from pooled replicates of young leaf, mature leaf, stem, flower and root tissue from 16-week-old *C. roseus* cv ‘Sunstorm Apricot’ plants grown in the greenhouse (Supplementary Table 4). RNA was isolated using the Qiagen Rneasy Plant Mini kit followed by mRNA isolation using the Dynabeads mRNA Purification Kit (Thermo Fisher Scientific, 61011). cDNA libraries were constructed using the ONT SQK-PCB109 kit and sequenced on R9 FLO-MIN106 Rev D flow cells; one library per tissue was constructed and sequenced on a single flow cell. ONT cDNA libraries were base-called using Guppy (v6.0.6 + 8a98bbc, nanoporetech.com/community) using the SUP model (dna_r9.4.1_450bps_sup.cfg) and the following parameters: --trim_strategy none --calib_detect. Base-called reads were processed with Pychopper (v2.5.0, github.com/nanoporetech/pychopper) to identify putative FL-cDNA reads, which were then aligned to the genome assembly using Minimap2 (ref. ^62^; v2.17-r941) with the following parameters: -a -x splice -uf -G 5000. Transcript assemblies were generated from the alignments using StringTie2 (ref. ^61^; v2.2.1).

Initial gene predictions were generated using the BRAKER^63^ (v2.1.5) pipeline using the RNA-seq Stringtie genome-guided alignments as transcript evidence. Gene predictions were refined using the RNA-seq and ONT cDNA transcript assemblies with two rounds of PASA2 (ref. ^64^; v2.4.1). MIA biosynthetic pathway genes were manually curated using WebApollo^65^ (v2.6.5). Functional annotation of the gene models was generated by searching the Arabidopsis proteome^66^ (TAIR10), Swiss-Prot plant proteins and PFAM^67^(v35) and assigning the function from the first informative match. Detection of paralogous MIA biosynthetic genes was performed using OrthoFinder^68^.

### Gene expression abundance estimations with bulk mRNA-seq samples

Publicly available *C. roseus* mRNA-seq datasets were downloaded from the National Center for Biotechnology Information SRA (Supplementary Table 4). Reads were cleaned using Cutadapt^59^ (v4.0) to trim adapters and remove low-quality sequences. Cleaned reads were aligned to the *C. roseus* genome (v3.0) using HISAT2 (ref. ^60^; v2.2.1) with a maximum intron size of 5 kb. Fragments per kilobase million (FPKM) were generated using Cufflinks^69^ (v2.2.1) with the following parameters: --multi-read-correct, --max-bundle-frags 999999999, --min-intron-length 20 and --max-intron-length 5000.

### Chromosome conformation capture and analyses methods

*C. roseus* cv ‘Sunstorm Apricot’ leaf tissue collected at the same time as one replicate of the 10x scRNA-seq experiments was used with the Proximo Hi-C kit (Phase Genomics; CRO_AR) to generate Hi-C reads and sequenced on the Illumina NovaSeq 6000 generating paired-end 150 nt reads. Fastq files were processed with Juicer^52^ and Juicer Tools v2.13.07 (ref. ^52^) to produce the .hic file (github.com/aidenlab/juicer/wiki). The inter30.hic output was used for all downstream analyses, which contains chromosome interactions using Q30 + reads. For loops, HiCCUPS (github.com/aidenlab/juicer/wiki/HiCCUPS) was used to detect chromosome loops at 5-kb resolution. For TAD domains, straw (github.com/aidenlab/straw) was used to access the data and write.txt files for each chromosome at 10-kb resolution. HiCkey (github.com/YingruWuGit/HiCKey) was used to detect TAD boundaries; all *P* values are corrected for multiple testing using the false discovery rate method.

### Protoplast isolation and scRNA-seq library generation

Protoplasts were isolated from young leaf tissue of 13–14-week-old plants from *C. roseus* cv ‘Sunstorm Apricot’ and used to generate scRNA-seq libraries using the 10x Chromium Controller (10x Genomics) and the Drop-Seq platform. Approximately 2 g of leaf tissue was used for protoplasting. For the 10x Genomics scRNA-seq, leaves were collected, and vacuum infiltrated with enzyme solution (0.4 M mannitol, 20 mM MES, 1.5% (wt/vol) cellulase (‘Cellulysin’; Sigma Aldrich, 219466), 0.3% (wt/vol) macerozyme R-10 (RPI, M22010), 1 mM calcium chloride, 0.1% BSA, pH 5.7) for 10 min at 400 mbar before being placed into a petri dish and shaken for 1 h and 45 min at 50 r.p.m. The plates were then shaken at 80–100 r.p.m. for 5 min to increase cell recovery. The resulting protoplast solution was filtered through a 40-µm mesh filter into a 50 ml tube with 5 ml of a storage solution (0.4 M mannitol, 20 mM MES, 1 mM calcium chloride, 0.1% bovine serum albumin, pH 5.7) used to rinse the plate and increase cell recovery. Protoplasts were gently pelleted at 150–200*g* for 3 min at 4 °C, and the supernatant was removed. The protoplasts were then gently resuspended in a storage solution to be counted and used for scRNA-seq library preparation, with additional filtering performed as needed. To generate a root single-cell expression dataset, ~5 g of roots from ~8-week-old plants were used and processed similarly to leaves.

A total of ~10,700 (leaf), ~2,600 (leaf), ~5,800 (leaf), ~3,400 (root) and ~4,000 (root) cells were used to generate 10x scRNA-seq libraries (Supplementary Table 12). In brief, the protoplast suspensions were loaded into a chromium microfluidic chip, and GEMs were generated using the 10x Chromium Controller (10x Genomics); libraries were constructed using the Single Cell 3′ v3.1 Kit (10x Genomics) according to the manufacturer’s instructions. For the Drop-Seq library, scRNA-seq protoplasts were isolated from young leaf tissue from *C. roseus* cv ‘Sunstorm Apricot’ and used to generate scRNA-seq libraries following the Drop-Seq method^24^ (mccarrolllab.org/download/905/). In brief, leaves were collected and protoplasts were generated as detailed above with the following modifications to the solutions: enzyme solution (0.6 M mannitol, 10 mM MES, 1.5% (wt/vol) cellulase (‘Cellulysin’; Sigma Aldrich, 219466), 0.3% (wt/vol) macerozyme R-10 (RPI, M22010), 1 mM calcium chloride, 0.1% BSA, pH 5.7) and storage solution (0.6 M, 10 mM MES, 1 mM calcium chloride, 0.1% bovine serum albumin, pH 5.7). In total ~115,000 protoplasts were run through the Drop-Seq protocol to generate the single-cell libraries. The 10x Genomics library SCP_AH was sequenced on a NextSeq 500 mid-output flow cell, and CRO_AS, CRO_AT, CRO_AW and CRO_AX were sequenced on a NextSeq 2000 P3 flow cell, with all runs sequenced as Read 1 at 28 nt and Read 2 at 91 nt and the index at 8 nt; in accordance with manufacturer recommendations. Drop-Seq libraries, CRO_AA and CRO_AB, were sequenced on three lanes of an Illumina MiSeq v3, with Read 1 being 25 nt and Read 2 being 100 nt.

### Single-cell transcriptome analysis

For 10x Genomics reads, Read 2 was cleaned using Cutadapt^59^ (v4.0) to remove adapters and poly-A tails; cleaned reads were then re-paired with Read 1. Cleaned reads were then aligned to a merged *C. roseus* genome (v3.0) and *C. roseus* chloroplast genome (NC_021423.1) using the STARsolo pipeline of STARsolo (v2.7.10)^70^ with the following parameters: --alignIntronMax 5000, --soloUMIlen 12, --soloCellFilter EmptyDrops_CR, --soloFeatures GeneFull, --soloMultiMappers EM, --soloType CB_UMI_Simple and --soloCBwhitelist using the latest 10× Genomics whitelist of barcodes.

Drop-Seq reads were processed in accordance with established Drop-Seq processing methods (github.com/broadinstitute/Drop-seq/blob/master/doc/Drop-seq_Alignment_Cookbook.pdf) using DropSeqTools (v2.5.1). Reads were trimmed using Cutadapt^59^ (v4.0) to remove adapters and poly-A tails and aligned to a merged *C. roseus* genome (v3.0) and *C. roseus* chloroplast genome (NC_021423.1) using HISAT2 (v2.2.1)^60^ with the parameters; --dta-cufflinks, --max-intronlen 5000 and --rna-strandness R. The Drop-Seq processing pipeline allows for various filtering approaches in how data is output; a minimum of 100 reads per cell cutoff was used to output our digital expression matrix.

### Removal of ambient RNA read counts

Four 10× libraries (CRO_AS, CRO_AT, CRO_AW and CRO_AX; Supplementary Table 12) were sequenced far deeper than the recommended 25,000 reads per cell, which led to the detection of higher background (that is, ambient) expression of cell-type-specific genes (Supplementary Fig. 19a). We used the R package DecontX^71^ to estimate and remove ambient RNA reads from the feature-count matrices of these four libraries. Median DecontX removed ambient reads per cell are reported in Supplementary Table 12; ambient reads-removed matrices for the abovementioned libraries were used for downstream analyses (Supplementary Fig. 19b).

### Cell type clustering

Drop-Seq and 10x Genomics expression matrices were loaded as Seurat objects^23^. Observations were filtered for between 200 and 3,000 RNA features and log normalized. Top 3,000 variable genes were selected for all runs and integrated using the ‘IntegrateData()’ function from Seurat. Uniform manifold approximation and projection (UMAP) were calculated using the first 30 principal components using the following parameters: ‘dims = 1:30, min.dist = 0.001, respulsion.strength = 1, n.neighbor = 30 and spread = 1.’ We curated a set of epidermis, mesophyll and vasculature marker genes for *C. roseus* (Supplementary Table 8) using orthologs^68^ of known markers from Arabidopsis^72,73^. For root cell markers, we curated markers from Arabidopsis^3,40,73,74^ and *C. roseus*^17^ (Supplementary Table 8). For single-cell gene expression heat maps (Figs. 4b and 6b and Extended Data Fig. 1b,c), average expression for each gene at each cell type is computed as the averaged *Z* scores for log-transformed normalized expression values, such that the color scale for each gene is relative to the mean and standard deviation of each gene across all cells. Dot sizes indicate the fraction of cells where a given gene is expressed (>0 reads) at a given cell type.

### Gene coexpression analyses

Pairwise correlation coefficients between the top 3,000 variable genes were computed using the ‘cor()’ function in R. A network edge table was produced from the correlation matrix, where each row is a gene pair. Statistical significance was calculated using a *t*-distribution approximation and adjusted for multiple testing using FDR. Only pairs with FDR < 0.01 were selected for downstream analysis. The network node table was constrained to known MIA biosynthetic enzymes and their first-degree network neighbors. Graphical representation of the network was produced using the ‘graph_from_data_frame()’ function from igraph^75^, using the filtered edge table and constrained network table as input. Network visualization was done using the ggraph package (ggraph.data-imaginist.com/).

### Leaf protoplast isolation for scMet analysis

Three leaves around 3.5 cm in length were selected. The leaves were cut into 1 mm strips with a sterile surgical blade. After that, the leaf strips were immediately transferred to a Petri dish with 10 ml of digestion medium (2% (wt/vol) cellulose Onozuka R-10, 0.3% (wt/vol) macerozyme R-10 and 0.1% (vol/vol) pectinase dissolved in mannitol/MES (MM) buffer. MM buffer contained 0.4 M mannitol and 20 mM MES, pH 5.7–5.8, adjusted with 1 M KOH. The open Petri dish was put inside a desiccator and a 100 mBar vacuum was applied for 15 min to infiltrate the medium into the leaf strips. The vacuum was gently disrupted for 10 s after every 1 min. The leaf strips were then incubated in the digestion medium for 2.5 h at room temperature. After the incubation, the Petri dish was placed on an orbital shaker at around 70 r.p.m., for 30 min at room temperature to help release the protoplasts. The protoplast suspension was filtered through a nylon sieve (70 μm) to remove larger debris and gently transferred to two 15 ml conical tubes. The protoplast suspension was centrifuged at 70*g* with gentle acceleration/deceleration, for 5 min, at 23 °C to pellet the protoplasts. The supernatant was removed as much as possible, and the protoplast pellet of each tube was washed three times by adding 5 ml of MM buffer, swirling gently and centrifuging. Finally, the last pellet from two tubes was pooled together and resuspended in 1 ml of MM buffer. The protoplast concentration was determined using a hemocytometer. The final concentration of protoplasts was adjusted to 10^6^ protoplasts in 1 ml.

### Cell picking for scMet analysis

A SIEVEWELL chip (ASL) with 90,000 nanowells (50 μm × 50 μm, depth × diameter) was used for single-cell trapping and sorting. The SIEVEWELL chip was primed with 100% ethanol, and then 1 ml of DPBS was immediately added to the chamber and then discarded through the side ports for washing. This washing step was repeated two times. After that, the chip was coated by carefully adding 1 ml (1.5%) BSA–DPBS and subsequently discarding the liquid through the side port. Then, MM buffer was added to replace the 1.5% BSA in the DPBS solution. Finally, 1 ml of protoplast suspension was carefully added and dispensed in a z-shape across the well. One milliliter of liquid was then discarded from the side ports.

The SIEVEWELL was then mounted on the CellCelector Flex (ALS Automated Lab Solutions) instrument, and the cells were visualized using the optical unit, constituted by a fluorescence microscope (Spectra X Lumencor) and a CCD camera (XM-10). Photos in transmitted light were acquired to cover all the chip. Single protoplasts were picked using a 50 μm glass capillary and dispensed into 96-well plates containing 5 μl of 0.1% formic acid. Pictures of the nanowells before (with single cell) and after picking (without single cell) were recorded. Cells were dried overnight and frozen at −20 °C until the analysis.

### scMet method

The UPLC–MS analysis was performed on a Vanquish (Thermo Fisher Scientific) system coupled to a Q-Exactive Plus (Thermo Fisher Scientific) orbitrap mass spectrometer. For metabolite separation, a Phenomenex Kinetex XB-C18 100 Å column (50 × 1.0 mm, 2.6 µm) was used at a temperature of 40 °C. The binary mobile phases were 0.1% HCOOH in MilliQ water (aqueous phase) (A) and acetonitrile (ACN) (B). The gradient elution started with 99% of the aqueous phase and increased with the ACN phase to 70% in 5.5 min, followed by an increase to 100% in the next 0.1 min. The percentage of ACN was held at 100% for 2 min before switching back to 99% of the water phase in 0.1 min. Finally, ACN was kept at 1% for 2.5 min to condition the column for the next injection. The total time for chromatographic separation was 12 min. In total, 4 μl of standards or samples were injected into the column via the autosampler. The flow rate of the mobile phase was kept constant at 0.3 ml min^−^^1^ during the chromatographic separation. Both samples and standard solutions were kept at 10 °C in the sample tray. The needle in the autosampler was washed using a mixture of methanol and MilliQ water (1:1, vol:vol) for 20 s after the draw and at a speed of 50 µl s^−1^.

The mass spectrometer was equipped with a heated electrospray ionization source. The mass spectrometer was calibrated using the Pierce positive and negative ion mass calibration solution (Thermo Fisher Scientific). The operating parameters of heated electrospray ionization are based on the UPLC flow rate of 300 µl min^−1^ using source auto default—sheath gas flow rate at 48; auxiliary gas flow rate at 11; sweep gas flow rate at 1; spray voltage +3500 V; capillary temperature at 250 °C; auxiliary gas heater temperature at 300 °C and S-lens RF level at 50. Acquisition was performed in full-scan MS mode (resolution 70000-FWHM at 200 Da) in positive mode over the mass range *m/z* from 120 to 1,000. The full MS/dd-MS2 (full-scan and data-dependent MS/MS mode) was used to simultaneously record the MS/MS (fragmentation) and the spectra for the precursors of QC pooled samples. The full MS/dd-MS2 (that included target analytes) was also used for QC pooled sample to confirm fragments of the selected precursors. The dd-MS2 was set up with the following parameters: resolution 35,000 FWHM; mass isolation window 0.4 Da; maximum and minimum automatic gain control target 8 × 10^3^ and 5 × 10^3^, respectively; normalized collision energy was set at three levels 15%, 30% and 45% and spectrum data format was centroid. All the parameters of the UPLC-MS system were controlled through Thermo Fisher Scientific Xcalibur software version 4.3.73.11 (Thermo Fisher Scientific). Chromatography and MS responses were optimized using several reference compounds (Supplementary Tables 10 and 11).

### Preparation of cells and QC samples

Before the analysis, the single cells were resuspended with 12 µl of 0.1% formic acid containing 10 nM of ajmaline as an internal standard. For pooled QC samples, 2 µl of sample from each well was taken and pooled together. For QC, 20 µl of *C. roseus* leaf protoplasts were extracted with 500 µl of pure MeOH. After sonication (10 min) and vortexing, the protoplast extract was filtered, diluted 200-fold with 0.1% formic acid containing 10 nM of ajmaline and used as a QC run. For QC total, we used a methanolic extract of one of the leaf strips used for making protoplasts. The tissue was ground to a fine powder using a TissueLyser II (Qiagen). Metabolites were extracted from the powdered leaf sample with 300 µl of pure MeOH. After vortexing and sonication for 10 min, the leaf extracts were filtered, and 5 µl aliquots were placed into Eppendorf tubes and dried under vacuum. Before analysis, one Eppendorf tube was taken out, resuspended in 1 ml of the extraction solution (0.1% formic acid containing 10 nM of ajmaline), sonicated for 10 min, filtered and used as a QC total.

### Preparation of standard solutions and calibration curves

Catharanthine (Sigma Aldrich), vindoline (Chemodex), serpentine hydrogen tartrate (Sequoia Research Products), anhydrovinblastine disulfate (Toronto Research Chemicals) and vinblastine sulfate (Sigma Aldrich) were dissolved in pure MeOH at a concentration of 10 µM. Serial dilutions (*n* = 15) were made between 0.1 nM and 1,000 nM and analyzed by UPLC–MS. The extracted peak areas were used to calculate linear regression curves (Supplementary Table 10).

### XCMS analysis and statistical analysis

Peak detection was performed using the XCMS^76^ centWave^77^ algorithm with a prefilter intensity threshold of 5 × 10^4^ counts, a maximum deviation of 3 ppm, a signal-to-noise ratio greater than 5 and peak width from 5 to 30 s, integrating on the real data. Peaks were grouped using a density approach with a bandwidth of 1, retention time was corrected with a locally estimated scatterplot smoothing (loess) and a symmetric fit (Tukey’s biweight function), and peaks were regrouped after correction. Finally, gap-filling was performed by integrating raw data in each peak group region. We used CAMERA^78^ to group redundant features (isotopes, adducts and in-source fragments) taking the injections of the QCs of the pooled samples as representative runs. Peaks were grouped within a window of 50% of the full width at half maximum (FWHM); isotopes were detected for single and doubly charged species with an error of 1 ppm; pseudospectra were grouped within sample correlation of extracted ion chromatograms, with a correlation threshold of 0.85 and a *P* value threshold of 0.05; and finally, adducts were determined for single cluster ions with 1 ppm error.

Only one representative feature for each correlation group was selected, with preference given to the peaks detected in the largest number of single-cell samples, and breaking ties by the total sum of intensities. To reduce variation due to injection, we scaled raw areas by the recovery of the internal standard (ajmaline) in each run. Artifacts were removed by keeping only features that were detected in all injection batches, and the ajmaline-corrected, log-transformed areas were centered and scaled on a per-batch basis, to minimize the effect of batch-to-batch variation. PCA was performed on this matrix using the *base* library of the R programming language (v4.1.3).

We assigned the identities of detected features by searching the exact mass within the limits of the feature *m/z* as detected by XCMS, and the retention time within 5 s of the experimental elution time of the standard, and we manually verified the assignations by comparing the QC runs against an injection of a mix of standards that was performed at the beginning and end of each batch.

### VIGS of *SLTr* transporter, D4H, THAS1, THAS2 and THAS1/2

A 516 bp fragment of the *SLTr* transporter was amplified from *C. roseus* Sunstorm Apricot leaves cDNA using the primers reported in Supplementary Table 13 and cloned into the pTRV2u vector as previously described^32^. A 300 bp fragment of the *THAS1* gene was PCR amplified from *C. roseus* genomic DNA (gDNA), while two 300 bp fragments from the same region of the *THAS2* gene with BamHI or SalI 5′ restriction overhangs, respectively, were PCR amplified from *C. roseus* cDNA. For the THAS1 and THAS2 double construct, the *THAS1* gene fragment was cut with XhoI and ligated to the THAS1 PCR fragment with the SalI overhang cut with the same enzyme. The four obtained fragments carrying BamHI and XhoI restriction overhangs were cloned in the BamHI and XhoI digested VIGS vector pTRV2-MgChl (pTRV2-ChlH(F1R2) from ref. ^79^) using the In-Fusion Snap Assembly Master Mix (Clontech Takara), yielding plasmids pTRV2-THAS1, pTRV2-THAS2 and pTRV2-THAS1/2, respectively. A 234 bp fragment of the *D4H* gene was amplified from cDNA and ligated into the pTRV2-MgChl using the In-Fusion Snap Assembly Master Mix (Clontech Takara), yielding plasmids pTRV2-D4H. The primer sequences used for cloning are given in Supplementary Table 12. Genomic DNA from *C. roseus* was isolated using the DNeasy Plant Mini Kit (Qiagen), and total RNA was isolated using the RNeasy plant Mini kit (Qiagen) and converted to cDNA using SuperScript IV VILO reverse transcriptase (Thermo Fisher Scientific). PCR was performed using Phusion DNA Polymerase (Thermo Fisher Scientific) according to the instructions of the manufacturer. All restriction enzymes were from New England BioLabs.

*Agrobacterium tumefaciens* GV3101 electrocompetent cells (Goldbio, CC-207) were transformed with the construct by electroporation. Agrobacterium GV3101 cells containing pTRV1 and the pTRV2 constructs were grown overnight in 5 ml LB supplemented with rifampicin, gentamycin and kanamycin at 28 °C. Cultures were pelleted at 3,000*g*, resuspended in inoculation solution (10 mM MES, 10 mM MgCl_2_ and 200 µM acetosyringone) to an OD600 of 0.7 and incubated at 28 °C for 2 h. Transformants were confirmed by PCR using the gene-specific primers used to amplify the gene fragment. Strains containing pTRV2 constructs were mixed 1:1 with pTRV1 culture, and this mixture was used to inoculate plants by the pinch wounding method. Plants (8–12 and 4 weeks old) were inoculated for each construct. Silenced leaves were collected 21–25 d postinoculation, ground in a TissueLyser II (Qiagen) and stored at −80 °C before analysis by quantitative PCR (qPCR) and UPLC–MS. qPCR was performed as reported previously^32^ using the primers in Supplementary Table 12.

For UPLC–MS of the VIGS tissue, 10–20 mg of frozen powder were extracted in 1:30 wt/vol of methanol containing 2 µM ajmaline as internal standard, sonicated for 10 min and incubated at room temperature for 1 h. After filtration through 0.2 µm polytetrafluoroethylene filters, the samples were diluted 1:10 with methanol before analysis by UPLC–MS. Samples were analyzed either on a Shimadzu IT-TOF or a Bruker Impact II qTOF instrument. Chromatography was performed on a Phenomenex Kinetex C18 XB (100 × 2.10 mm × 2.6 µm) column and the binary solvent system consisted of ACN and 0.1% formic acid in water. Compounds were separated using a linear gradient of 10–30% B in 5 min followed by 1.5 min isocratic at 100% B. The column was then re-equilibrated at 10% B for 1.5 min. The column was heated to 40 °C and flow rate was set to 0.6 ml min^−^¹.

### Protein expression and activity assays

Cloning of *THAS1* (KM524258.1) and *THS2* (KU865323.1) was described in ref. ^14^. *ADH20* (KU865330.1), *ADH32* (AYE56096.1) and *ADH92* (ON911573) genes were amplified from *C. roseus* ‘Sunstorm Apricot’ leaves cDNA using the primers reported in Supplementary Table 13. The sequences of *ADH20* and *ADH92* are reported in Supplementary Table 14. The PCR products were purified from agarose gel, ligated into the BamHI and KpnI restriction sites of the pOPINF vector^80^ using the In-Fusion kit (Clontech Takara) and transformed into chemically competent *E. coli* One-Shot Top10 cells (Thermo Fisher Scientific, C404010). Recombinant colonies were selected on LB agar plates supplemented with carbenicillin (100 μg ml^−^^1^). Positive clones were identified by colony PCR using T7_Fwd and pOPIN_Rev primers (Supplementary Table 13). Plasmids were isolated from positive colonies grown overnight. Identities of the inserted sequences were confirmed by Sanger sequencing. Chemically competent SoluBL21 *E. coli* cells (Amsbio) were transformed by heat shock at 42 °C. Transformed cells were selected on LB agar plates supplemented with carbenicillin (100 μg ml^−1^). Single colonies were used to inoculate starter cultures in 10 ml of 2× YT medium supplemented with carbenicillin (100 μg ml^−1^) that were grown overnight at 37 °C. Starter culture (1 ml) was used to inoculate 100 ml of 2× YT medium containing the antibiotic. The cultures were incubated at 37 °C until OD600 reached 0.6 and then transferred to 16 °C for 30 min before induction of protein expression by the addition of IPTG (0.2 mM). Protein expression was carried out for 16 h. Cells were extracted by centrifugation and resuspended in 10 ml of buffer A (50 mM Tris–HCl pH 8, 50 mM glycine, 500 mM NaCl, 5% glycerol, 20 mM imidazole,) with EDTA-free protease inhibitors (Roche Diagnostics). Cells were lysed by sonication for 2 min on ice. Cell debris was removed by centrifugation at 35,000*g* for 20 min. Ni-NTA resin (200 μl, Qiagen) was added to each sample and the samples were incubated at 4 °C for 1 h. The Ni-NTA beads were sedimented by centrifugation at 1500 *g* for 1 min and washed three times with 10 ml of buffer A. The enzymes were step-eluted using 600 µl of buffer B (50 mM Tris–HCl pH 8, 50 mM glycine, 500 mM NaCl, 5% glycerol, 500 mM imidazole) and dialyzed in buffer C (25 mM HEPES pH 7.5, 150 mM NaCl). Enzymes were concentrated and stored at −20 °C before in vitro assays.

To assay the activity of the ADHs, the substrate (AHVB iminium) first had to be generated in vitro. For this purpose, 500 µl reactions were assembled in 50 mM MES buffer (pH 6.5). Each reaction contained 100 µM vindoline, 600 µM catharanthine, 0.002% hydrogen peroxide and 22.5 U of horseradish peroxidase (Sigma Aldrich, 77332). The reactions were incubated at 30 °C for 45 min, after which the sample was divided into aliquots of 70 µl each. To each aliquot, NADPH was added to a concentration of 200 µM and the ADHs to a concentration of 1 µM. The final volume was 100 µl. The reactions were incubated for 2 h and 3 µl were taken at different time points to monitor the progression of the reactions. The 3 µl samples were quenched in 97 µl of MeOH, filtered and analyzed by UPLC–MS on a Thermo Ultimate 3000 chromatographic system coupled to a Bruker Impact II mass spectrometer. Separation was performed on a Phenomenex Kinetex 2.7 µm C18 100 Å (100 × 2.10 mm × 2.7 µm) column and the binary solvent system consisted of ACN and 0.1% formic acid in water. The elution program was as follows: time 0–1 min, 10% ACN; linear gradient to 30% ACN in 5 min; column wash at 100% ACN for 1.5 min and then re-equilibration to 10% ACN for 2.5 min. Flow rate was 600 µl min^−1^. Data were analyzed using the Bruker Data Analysis software. Quantification of the AHVB produced during the reactions was performed using external calibration curves and used to calculate the specific activity of the enzymes. The experiments were performed in triplicate.

### Reporting summary

Further information on research design is available in the Nature Portfolio Reporting Summary linked to this article.

## Online content

Any methods, additional references, Nature Portfolio reporting summaries, source data, extended data, supplementary information, acknowledgements, peer review information; details of author contributions and competing interests and statements of data and code availability are available at 10.1038/s41589-023-01327-0.

## Supplementary information

Supplementary Information Supplementary Figs. 1–19.

Reporting Summary

Supplementary Tables Supplementary Tables 1–14.

## Data Availability

Data supporting the findings of this research are available within the Article, Extended Data, Source Data, Supplementary Tables and Supplementary Information. Sequences of the genes *THAS1* (KM524258.1), *THAS2* (KU865323.1), *ADH20* (KU865330.1), *ADH32* (AYE56096.1) and *ADH92* (ON911573) are available from Genbank. All sequencing data associated with this study are available at the National Center for Biotechnology Institute Short Read Archive BioProject PRJNA847226. Large files including the gene expression abundances from the bulk mRNA-seq, leaf and root scRNA-seq, genome assembly and genome annotation are available via the Dryad Digital Repository under 10.5061/dryad.d2547d851. Source data are provided in this paper.

## Source data

Source Data Fig. 2 (1) Fig. 2b: Genome assembly statistics. (2) Fig. 2c-1: Number of genes in 1-Mb bins along chromosomes. (3) Fig. 2c-2: Number of repetitive elements in 1-Mb bins along chromosomes.
Source Data Fig. 3 (1) Fig. 3a-1: Number of Hi-C contacts at 10-kb resolution. (2) Fig. 3a-2: Coordinate and annotation of genes. (3) Fig. 3a-3: *P* values of TAD boundaries. (4) Fig. 3b-1: Metabolite abundances of VIGS and control samples. (5) Fig. 3b-2: *t* statistics and *P* values for comparisons. (6) Fig. 3c-1: Number of Hi-C contacts at 10-kb resolution. (7) Fig. 3c-2: Coordinate and annotation of genes. (8) Fig. 3c-3: Coordinates of loops. (9) Fig. 3c-4: *P* values of TAD boundaries. (10) Fig. 3d-1: Number of Hi-C contacts at 10-kb resolution. (11) Fig. 3d-2: Coordinate and annotation of genes. (12) Fig. 3d-3: *P* values of TAD boundaries. (13) Fig. 3e: FPKM values.
Source Data Fig. 4 (1) Fig. 4a: Coordinates of cells in UMAP. (2) Fig. 4b-1: FPKM values of MIA pathway genes for bulk RNA-seq. (3) Fig. 4b-2: Average expression values and percent expressed for MIA pathway genes across cell types for scRNA-seq. (4) Fig. 4c-1: Edge table of network diagram. (5) Fig. 4c-2: Node table of network diagram.
Source Data Fig. 5 (1) Fig. 5b-1: PCA coordinates for scMet with secologanin *Z* score. (2) Fig. 5b-2: PCA coordinates for scMet with serpentine *Z* score. (3) Fig. 5c: Retention time and normalized ion intensity. (4) Fig. 5d-1: Estimated metabolite abundances for metabolites in the millimolar range. (5) Fig. 5d-2: Estimated metabolite abundances for metabolites in the micromolar range.
Source Data Fig. 6 (1) Fig. 6b: Average expression values and percent expressed for selected biosynthetic genes across cell types for scRNA-seq. (2) Fig. 6c-1: Specific activities of ADH candidates. (3) Fig. 6c-2: Means and confidence intervals of specific activities. (4) Fig. 6d: *Z* score of metabolite abundances across single cells.
Source Data Extended Data Fig. 1 (1) Extended Data Fig. 1a: Coordinates of cells in UMAP. (2) Extended Data Fig. 1b: Average expression values and percent expressed for MIA pathway genes across cell types for scRNA-seq in root. (3) Extended Data Fig. 1c-1: Average expression values and percent expressed for select paralogs across cell types for scRNA-seq in leaf. (4) Extended Data Fig. 1c-2: Average expression values and percent expressed for select paralogs across cell types for scRNA-seq in leaf.

## Code availability

All custom codes used to generate figures can be found at https://github.com/cxli233/Catharanthus_scRNA_seq.
