# Supplementary material for: Single-cell multi-omics in the medicinal plant Catharanthus roseus
Source: Nat Chem Biol. 2023 May 15;19(8):1031–41. doi: 10.1038/s41589-023-01327-0 (PMC10374443; doi:10.1038/s41589-023-01327-0)
Supplement: Supplementary file 1 — Supplementary Figs. 1–19. [file 41589_2023_1327_MOESM1_ESM.pdf]

nature chemical biology

Article

https://doi.org/10.1038/s41589-023-01327-0

Single-cell multi-omics in the medicinal plant *Catharanthus roseus*

In the format provided by the authors and unedited

SUPPLEMENTARY INFORMATION CONTENTS:

Supplemental Figure 1: Major branches of MIA pathway in *C. roseus* leaves

Supplemental Figure 2: Major branches of MIA pathway in *C. roseus* roots

Supplementary Fig. 3: Summary of adaptive finishing experiments

Supplementary Figure 4: Virus-induced gene silencing of *SLtr* in *C. roseus*

Supplementary Fig. 5: UPLC/MS analysis of *C. roseus* leaves

Supplementary Fig. **6:** Dimension reduction for leaf scRNA-seq

Supplementary Fig. 7: Comparison between 10x and Drop-seq platforms

Supplementary Fig. 8: Workflow for single cell mass spectrometry (scMS)

Supplementary Fig. 9: Extracted ion chromatograms of MIAs detected in a typical idioblast cell

Supplementary Fig. 10: MS/MS comparison between MIAs identified in the scMS experiment and available standards

Supplementary Fig. 11: Relative comparison of three alkaloids in the leaf tissue extract and in the protoplasts extract

Supplementary Fig. 12: MS/MS comparison between MIAs identified in the scMS experiment and available standards

Supplementary Fig. 13: Quantification of MIAs in single idioblasts

Supplementary Fig. 14: Detection of AHVB iminium in an idioblast cell

Supplementary Fig. 15: Virus-induced gene silencing of D4H, THAS1, THAS2 and of the THAS1/2 co-silencing in *C. roseus*

Supplementary Fig. 16: Metabolite analysis of tissue that has been transformed with a VIGS vector that targets D4H, THAS1, THAS2 and both THAS1/2 relative to the empty vector (EV) control

Supplementary Fig. **17:** Dimension reduction and cell type assignment for root scRNA-seq data

Supplementary Fig. **18:** Expression of selected MIA biosynthetic genes in the root

Supplementary Fig. **19:** Dot plots showing expression of MIA biosynthetic gene across cell type

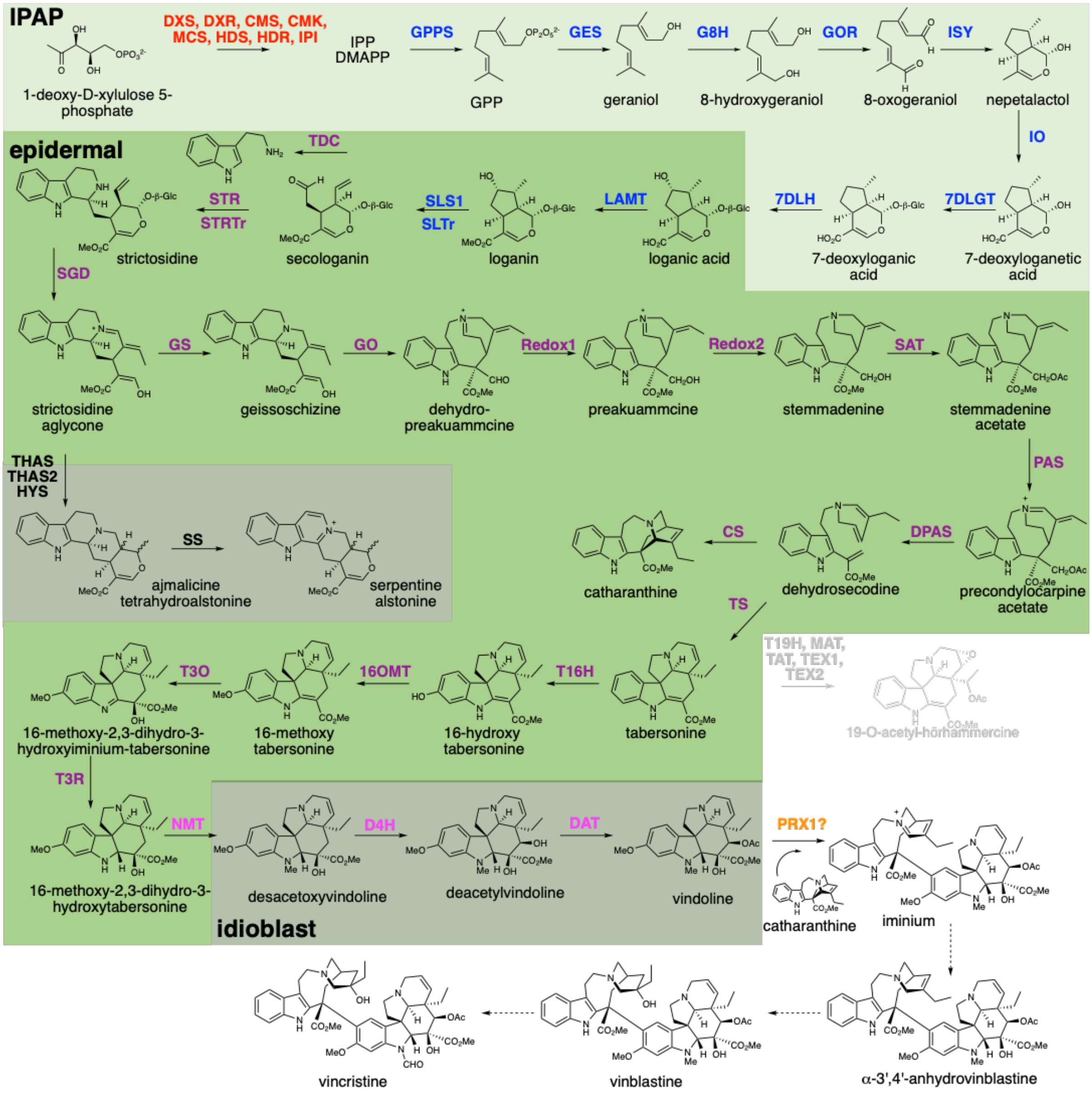

Supplemental Figure 1: Major branches of MIA pathway in *C. roseus* leaves. MEP pathway enzymes are shown in red; iridoid pathway enzymes are shown in blue; alkaloid pathway enzymes are shown in purple; late alkaloid pathway enzymes are shown in pink. The putative peroxidase PRX1 that couples catharanthine and vindoline is shown in orange. Enzymes leading to heteroyohimbines are shown in black. Root specific pathway enzymes that derivatize tabersonine are shown gray. Localization in specific leaf cell types are indicated by transparent green boxes: light green represents IPAP cells, medium green represents epidermal cells, dark green represents idioblast cells. Dashed arrows indicate steps for which biosynthetic genes have not been discovered.

Abbreviations: *DXS*: 1-deoxy-D-xylulose 5-phosphate synthase, *DXR*: 1-deoxy-D-xylulose-5-phosphate reductoisomerase, *CMS*: 4-diphosphocytidyl-methylerythritol 2-phosphate synthase, *CMK*: 4-diphosphocytidyl-2-C-methyl-D-erythritol kinase, *MCS*: 2C-methyl-D-erythritol 2,4-cyclodiphosphate synthase, *HDS*: 1-hydroxy-2-methyl-2-(E)-butenyl-4-diphosphate synthase, *HDR*: 1-hydroxy-2-methyl-butenyl 4-diphosphate reductase, *IPI*:

plastid isopentenyl pyrophosphate:dimethylallyl pyrophosphate isomerase, *GPPS*: geranyl pyrophosphate synthase, *GES*: geraniol synthase, *G8H*: geraniol 8-hydroxylase, *GOR*: 8-hydroxygeraniol oxidoreductase, *ISY*: iridoid synthase, *IO*: iridoid oxidase, *7DLGT*: UDP-glucose iridoid glucosyltransferase, *7DLH*: 7-deoxyloganic acid 7-hydroxylase, *LAMT*: loganic acid methyltransferase, *SLS1*: secologanin synthase, *SLTr*: secologanin transporter, *TDC*: tryptophan decarboxylase, *STR*: strictosidine synthase, *STRTr*: strictosidine transporter, *SGD*: strictosidine β-glucosidase, *GS1*: geissoschizine synthase, *GO*: geissoschizine oxidase, *Redox1*: reductive enzyme 1, *Redox2*: reductive enzyme 2, *SAT*: stemmadenine acetyl transferase, *PAS*: precondylocarpine acetate synthase, *DPAS*: dihydroprecondylocarpine acetate synthase, *CS*: catharanthine synthase, *TS*: tabersonine synthase, *T16H2*: tabersonine 16-hydroxylase, *16OMT*: 16-hydroxytabersonine O-methyltransferase, *T3O*: tabersonine 3-oxidase, *T3R*: tabersonine 3-reductase, *NMT*: 16-hydroxy-2,3-dihydro-3-hydroxytabersonine N-methyltransferase, *D4H*: desacetoxyvindoline-4-hydroxylase, *DAT*: deacetylvindoline 4-O-acetyltransferase, *THAS, THAS2*: tetrahydroalstonine synthase, *HYS*: heteroyohimbine synthase, *SS*, serpentine synthase, *TEX1,2*: tabersonine 6,7-epoxidase, *T19H*: tabersonine/lochnericine 19-hydroxylase, *TAT*: tabersonine derivative 19-O-acetyltransferase, *MAT*: minovincinine 19-hydroxy-O-acetyltransferase.

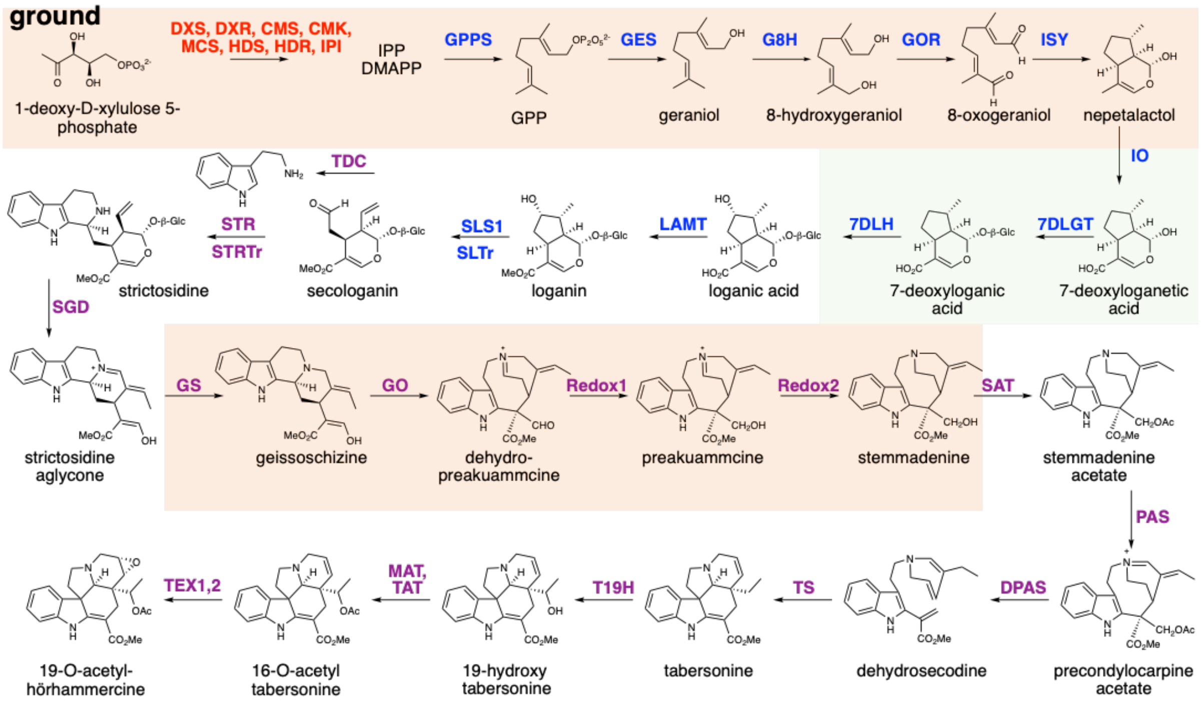

Supplemental Figure 2: Major branches of MIA pathway in *C. roseus* roots. MEP pathway enzymes are shown in red; iridoid pathway enzymes are shown in blue; alkaloid pathway enzymes are shown in purple. In roots, the pathway is not partitioned into three discrete cell types as in leaves. Instead, the MEP and iridoid stages are specifically expressed in the ground tissue composed of the cortex and endodermis with expression of the alkaloid stage, while also expressed in ground tissues, exhibiting a more diffused expression pattern. Abbreviations: *DXS*: 1-deoxy-D-xylulose 5-phosphate synthase, *DXR*: 1-deoxy-D-xylulose-5-phosphate reductoisomerase, *CMS*: 4-diphosphocytidyl-methylerythritol 2-phosphate synthase, *CMK*: 4-diphosphocytidyl-2-C-methyl-D-erythritol kinase, *MCS*: 2C-methyl-D-erythritol 2,4-cyclodiphosphate synthase, *HDS*: 1-hydroxy-2-methyl-2-(E)-butenyl-4-diphosphate synthase, *HDR*: 1-hydroxy-2-methyl-butenyl 4-diphosphate reductase, *IPI*: plastid isopentenyl pyrophosphate:dimethylallyl pyrophosphate isomerase, *GPPS*: geranyl pyrophosphate synthase, *GES*: geraniol synthase, *G8H*: geraniol 8-hydroxylase, *GOR*: 8-hydroxygeraniol oxidoreductase, *ISY*: iridoid synthase, *IO*: iridoid oxidase, *7DLGT*: UDP-glucose iridoid glucosyltransferase, *7DLH*: 7-deoxyloganic acid 7-hydroxylase, *LAMT*: loganic acid methyltransferase, *SLS1*: secologanin synthase, *SLTr*: secologanin transporter, *TDC*: tryptophan decarboxylase, *STR*: strictosidine synthase, *STRTr*: strictosidine transporter, *SGD*: strictosidine β-glucosidase, *GS1*: geissoschizine synthase, *GO*: geissoschizine oxidase, *Redox1*: reductive enzyme 1, *Redox2*: reductive enzyme 2, *SAT*: stemmadenine acetyl transferase, *PAS*: precondylocarpine acetate synthase, *DPAS*: dihydroprecondylocarpine acetate synthase, *TS*: tabersonine synthase, *TEX1,2*: tabersonine 6,7-epoxidase, *T19H*: tabersonine/lochnericine 19-hydroxylase, *TAT*: tabersonine derivative 19-O-acetyltransferase, *MAT*: minovincinine 19-hydroxy-O-acetyltransferase.

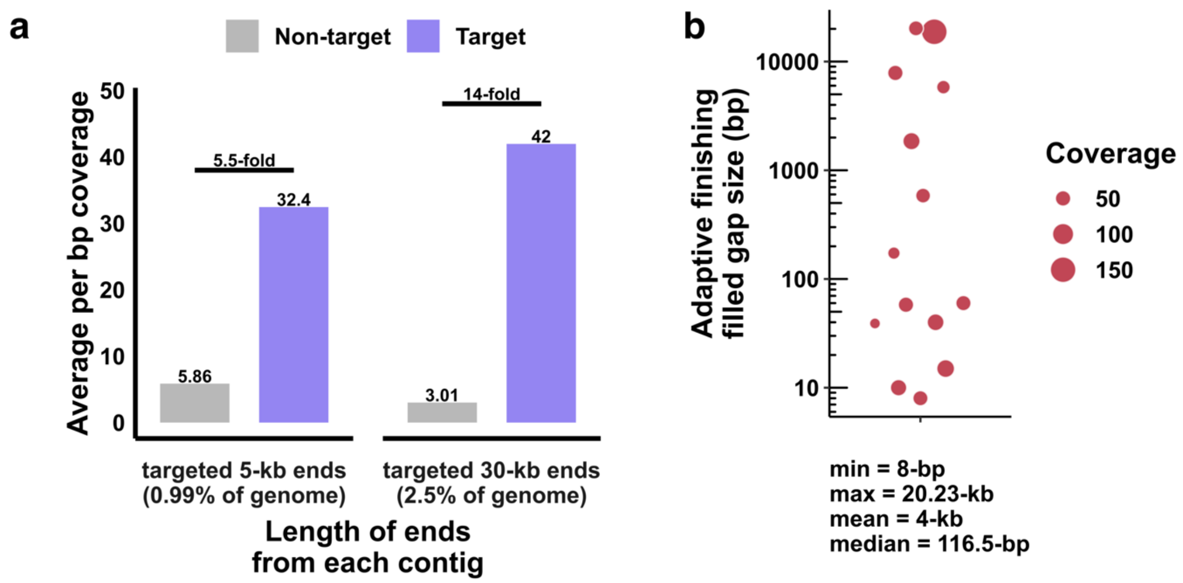

Supplementary Fig. 3: Summary of adaptive finishing experiments. **a**, Adaptive sampling of the *C. roseus* genome in which the terminal 5-kb ends of contigs (left) or the terminal 30-kb ends of contigs (right) were used in the adaptive sampling. **b**, Coverage and filled gap size of the adaptive sampling.

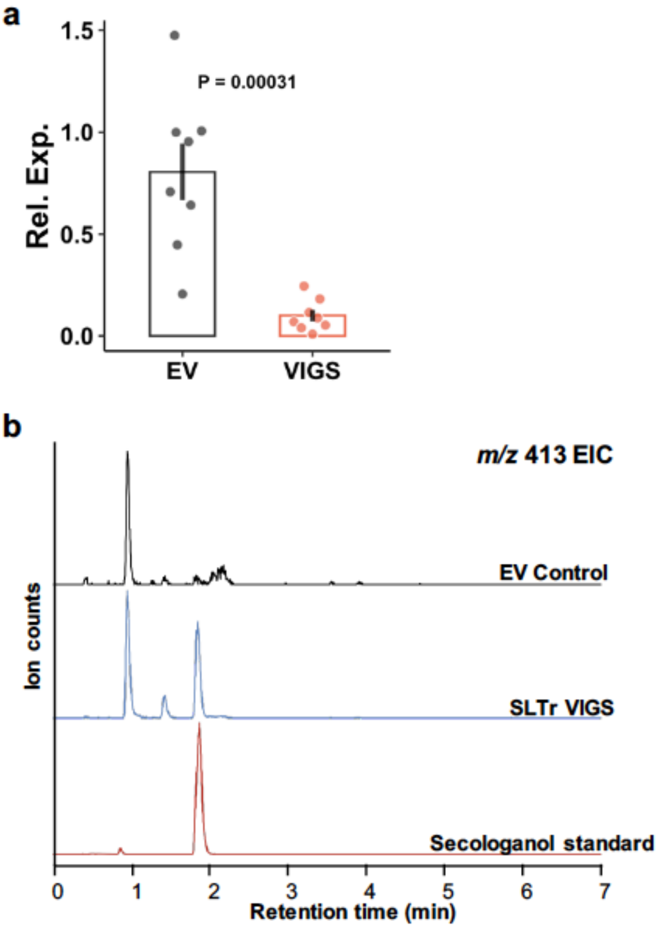

Supplementary Figure 4: Virus-induced gene silencing of *SLtr* in *C. roseus*. a, normalized relative expression of the silenced gene (VIGS) compared to the empty vector (EV) control plants as measured by qPCR. Values were calculated using 2-**ΔΔ**Ct and using Rps9 as reference gene. 8 biological replicates for control (EV) and silenced plants were used (n=8). Data are presented as mean values +/- SEM. Unpaired, two-tailed t-test was used for comparisons and the P value is shown in the graph. **b**, extracted ion chromatograms of *m/z* 413 accumulating in *C. roseus* leaves after silencing the *SLTr* gene. This peak is clearly not present in the empty vector (EV) control leaves. Co-elution with a standard of secologanol produced by chemical reduction of secologanin with NaBH4 confirmed that the peak at *m/*z 413 $[M+Na]^+$ is indeed secologanol.

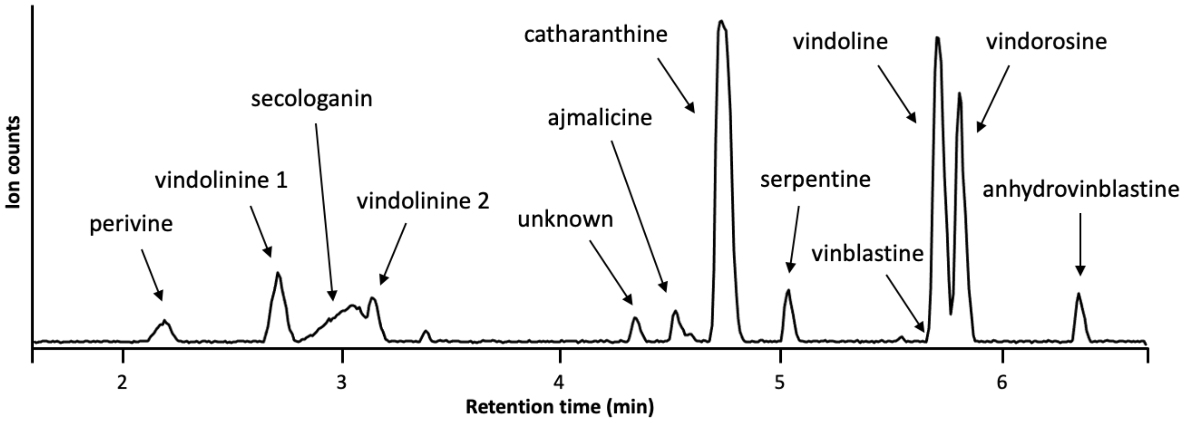

Supplementary Fig. 5: UPLC/MS analysis of *C. roseus* leaves. Total ion chromatogram of a methanolic extract of the leaves used for the scRNA-seq experiments showing the occurrence of the most abundant alkaloids.

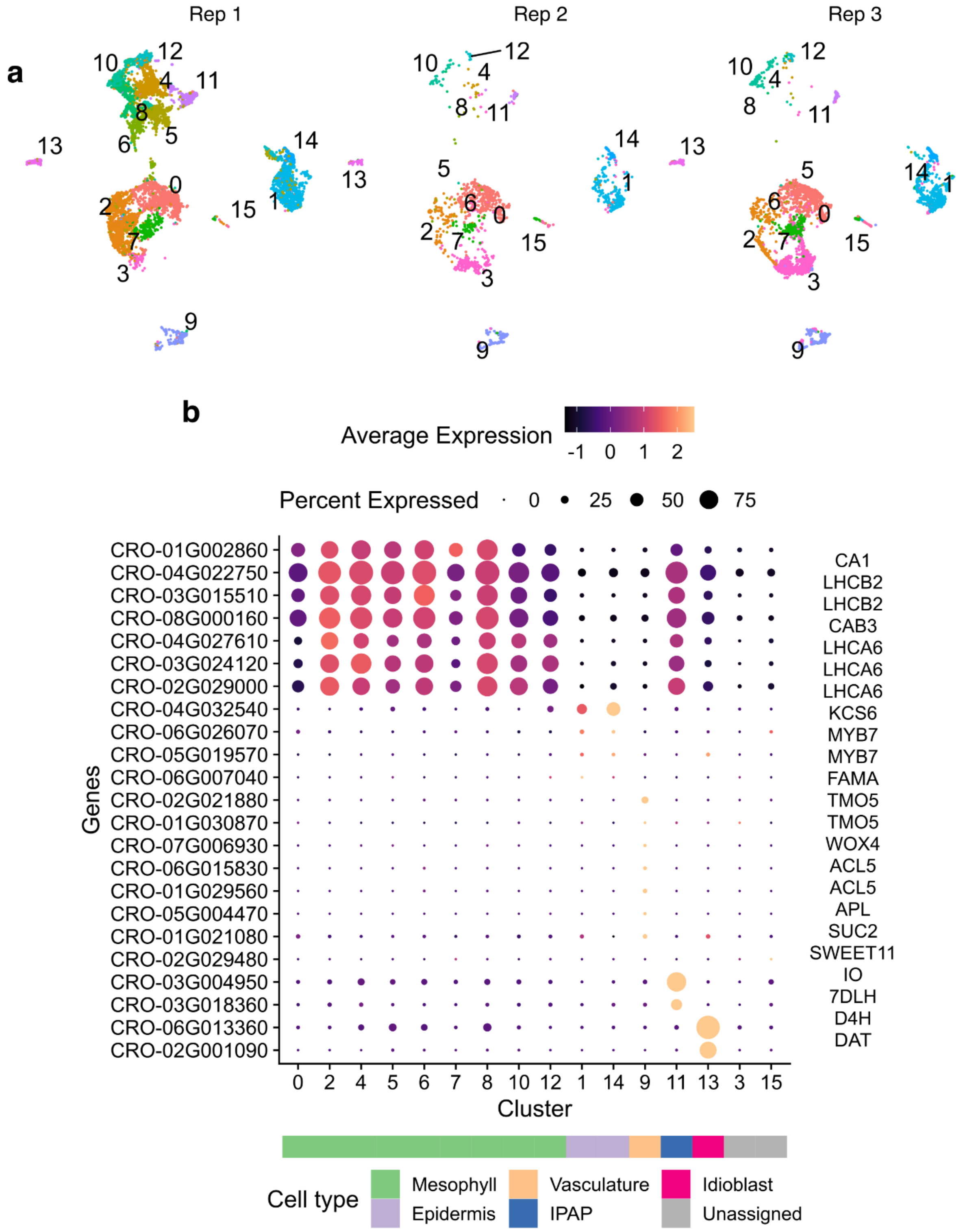

Supplementary Fig. 6: Dimension reduction for leaf scRNA-seq. **a**, UMAP (uniform manifold approximation and projection) for *C. roseus* leaves; subpanels grouped by biological replicates. **b**, Cell type assignment for leaf scRNA-seq dataset. Each row is a marker gene (see also Supplemental Table 8). v3 annotation gene IDs are on the left; Arabidopsis ortholog gene names on the right. Dot color indicates expression level. Dot size indicates the percentage of cells expressing the marker.

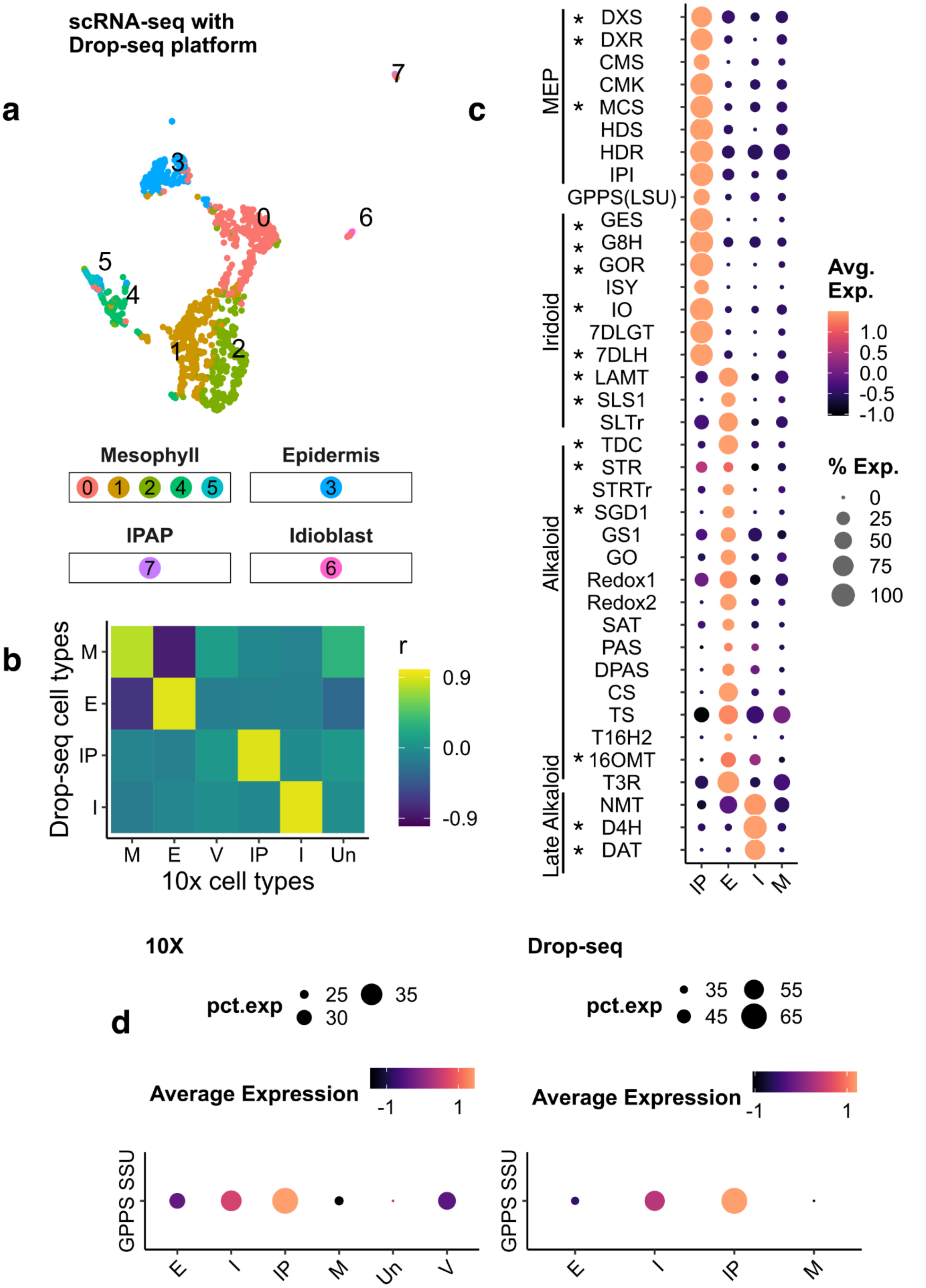

scRNA-seq with Drop-seq platform
a
b
c
d
Mesophyll
0 1 2 4 5
Epidermis
3
IPAP
7
Idioblast
6
Drop-seq cell types
M
E
IP
I
10x cell types
M E V IP I Un
r
0.9
0.0
-0.9
MEP
Iridoid
Alkaloid
Late Alkaloid
* DXS
* DXR
CMS
CMK
* MCS
HDS
HDR
IPI
GPPS(LSU)
* GES
* G8H
* GOR
ISY
* IO
7DLGT
* 7DLH
* LAMT
* SLS1
SLTr
* TDC
* STR
STRTr
* SGD1
GS1
GO
Redox1
Redox2
SAT
PAS
DPAS
CS
TS
T16H2
* 16OMT
T3R
NMT
* D4H
* DAT
IP E I M
Avg. Exp.
1.0
0.5
0.0
-0.5
-1.0
% Exp.
0
25
50
75
100
10X
pct.exp
25 30 35
Average Expression
-1 1
GPPS SSU
E I IP M Un V
Drop-seq
pct.exp
35 45 55 65
Average Expression
-1 1
GPPS SSU
E I IP M

Supplementary Fig. 7: Comparison between 10x and Drop-seq platforms. a, UMAP (uniform manifold approximation and projection) for *C. roseus* leaves using the Drop-seq platform. **b**, Heat map comparing cell types recovered by the Drop-seq platform vs. the 10x platform. Color indicates correlation coefficient across the top 3000 variable genes. **c**, Gene expression heatmap of the MIA biosynthetic pathway for bulk and single cell transcriptomes. Genes are arranged from upstream to downstream. Previously reported cell type specific expression are confirmed and marked with *. T3O was not detected in the Drop-seq dataset. **d**. Dot plot showing geranyl diphosphate synthase small subunit (GPPS-SSU) expression across cell types in 10x and Drop-seq platform. See Supplementary Fig. 1, Supplementary Table 6 for list of gene name abbreviations. IP/ IPAP: internal phloem associated parenchyma; E: epidermis; I; idioblast; M: mesophyll; V: vasculature; Un: unassigned.

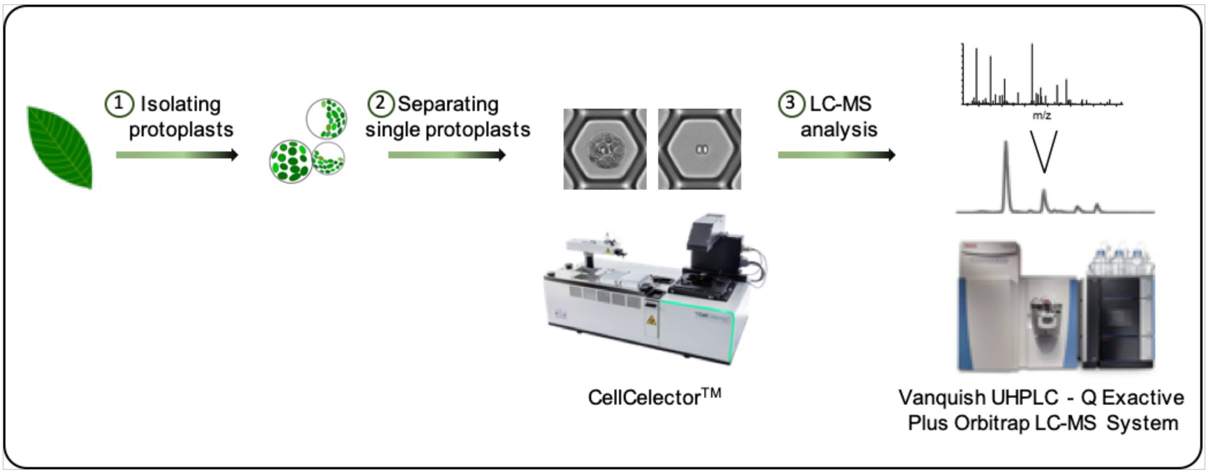

Supplementary Fig. 8: Workflow for single cell mass spectrometry (scMS). 1. Leaves are cut into strips and incubated in a mixture of enzymes to release the protoplasts. 2. The protoplast suspension is then applied onto a Sievewell chip for sorting using the CellCelector robot. 3. The single cells are transferred into 96-well plates, lysed and injected into the LC/MS system.

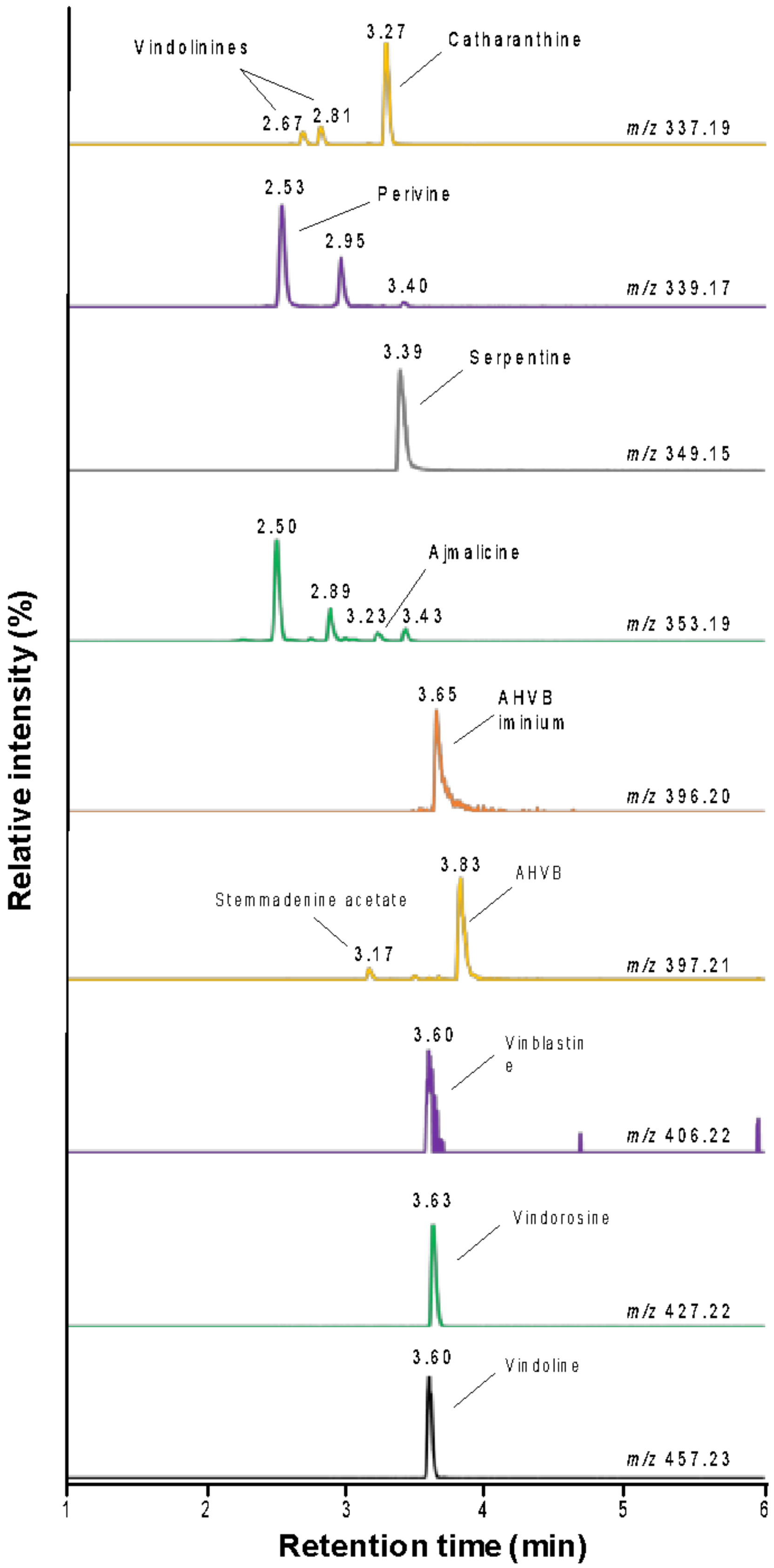

Supplementary Fig. 9: Extracted ion chromatograms of MIAs detected in a typical idioblast cell.

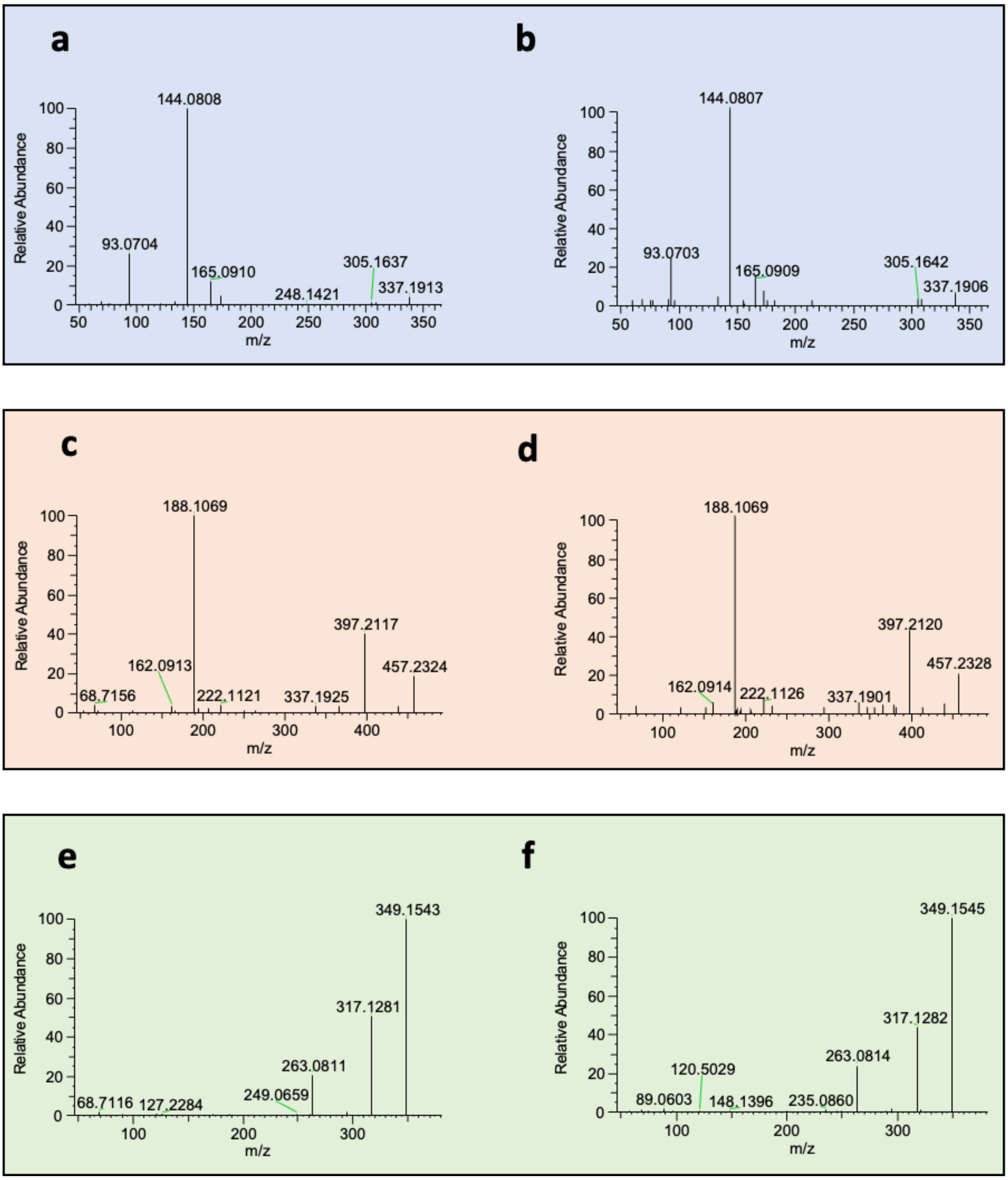

Supplementary Fig. 10: MS/MS comparison between MIAs identified in the scMS experiment and available standards. a, MS/MS spectrum of catharanthine detected in an idioblast cell. **b**, MS/MS spectrum of catharanthine standard. **c**, MS/MS spectrum of vindoline detected in an idioblast cell. **d**, MS/MS spectrum of vindoline standard. **e**, MS/MS spectrum of serpentine detected in an idioblast cell. **f**, MS/MS spectrum of serpentine standard.

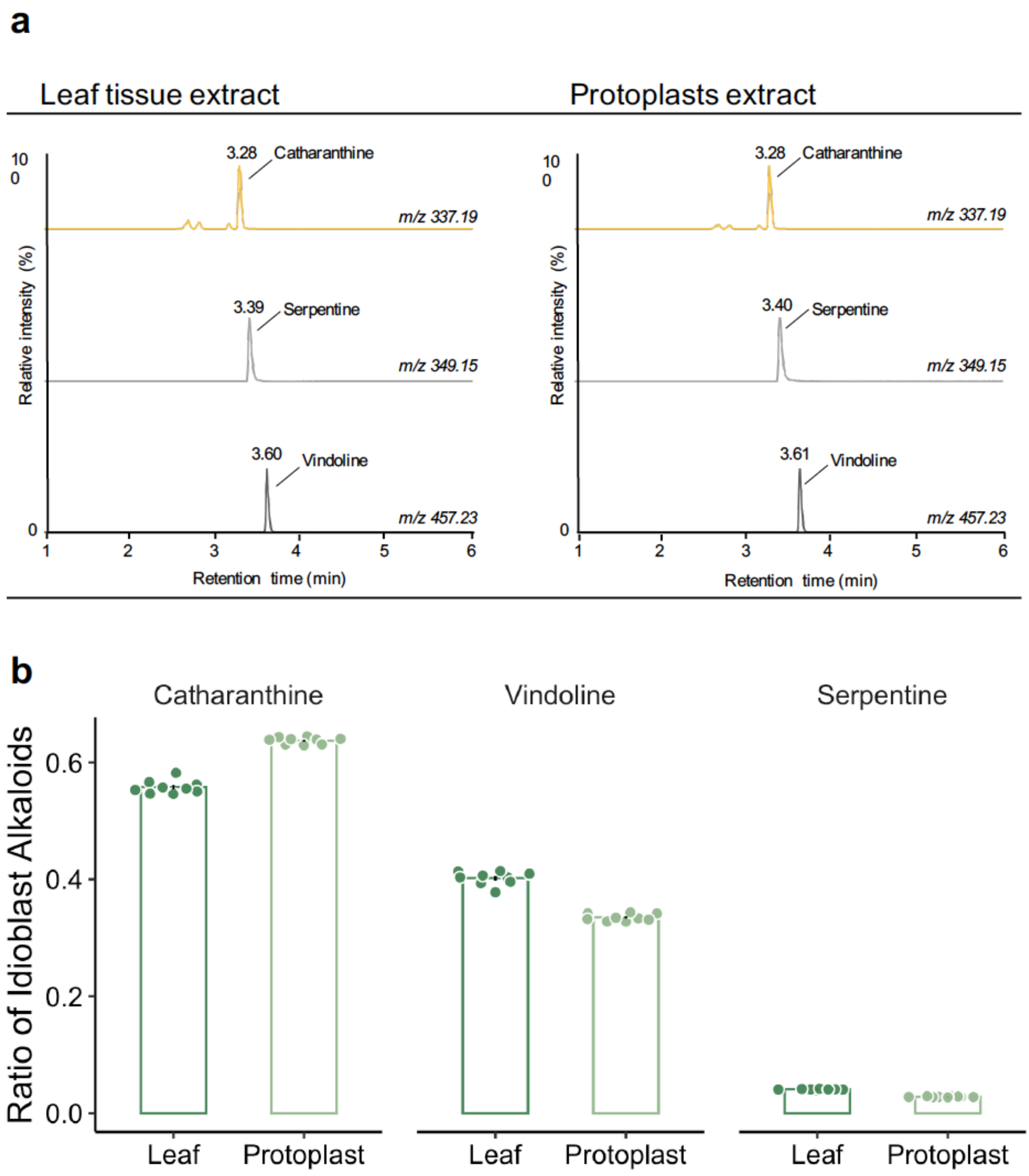

Supplementary Fig. 11: Relative comparison of three alkaloids in the leaf tissue extract and in the protoplasts extract. a, extracted ion chromatograms for the compounds catharanthine, serpentine and vindoline in leaf tissues used for producing protoplasts and the final protoplast preparation used for collecting single cells. **b**, comparison of the peak areas of the three compounds in the leaf tissue and the protoplast preparation normalized by to the total peak area of these compounds in the samples (n=9). Data are presented as mean values +/- SEM.

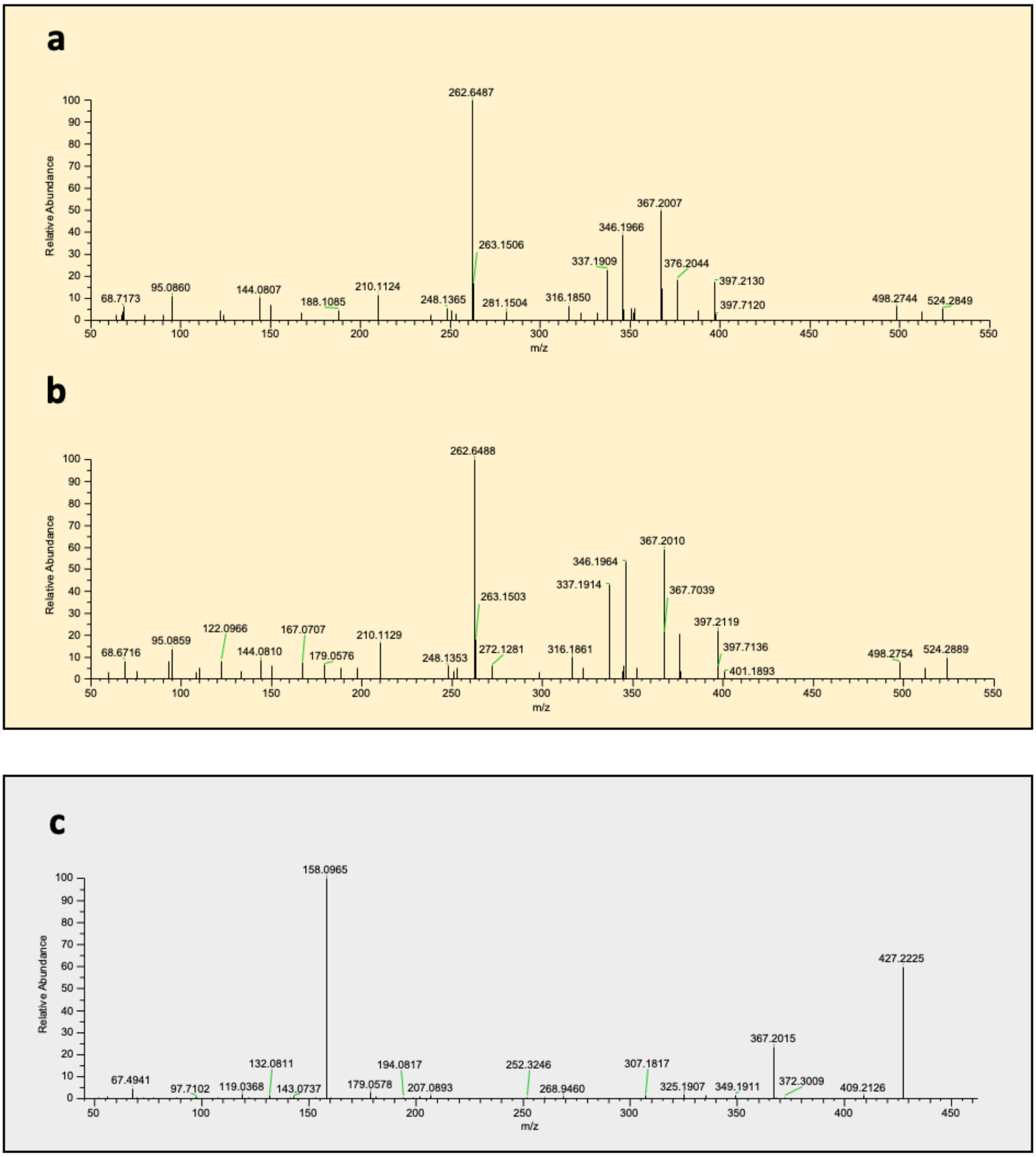

Supplementary Fig. 12: MS/MS comparison between MIAs identified in the scMS experiment and available standards. a, MS/MS spectrum of the bisindole anhydrovinblastine detected in an idioblast cell. **b**, MS/MS spectrum of anhydrovinblastine standard. **c**, MS/MS spectrum of vindorosine detected in a pooled QC sample. The spectrum is in agreement with that reported by Yamamoto *et al*. (2021).

Supplementary Fig. 13: Quantification of MIAs in single idioblasts.

	Image	Diameter [a] (µm)	Volume[b] (pL)	Catharanthine (mM) (fmol/cell)[c]	Vindoline (mM) (fmol/cell)[c]	Serpentine (mM) (fmol/cell)[c]	Vinblastine (µM) (fmol/cell)[c]	Anhydrovinblastine (µM) (fmol/cell)[c]
1	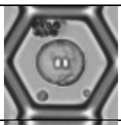	28.5	13	240 (3121)	19 (251)	16 (211)	-	142 (2)
2	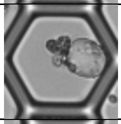	23.0	7	210 (1471)	25 (173)	5 (36)	-	118 (1)
3	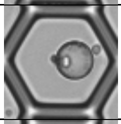	23.4	7	219 (1530)	5 (38)	19 (135)	-	270 (2)
4	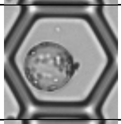	30.2	15	185 (2778)	6 (88)	7 (105)	-	209 (3)
5	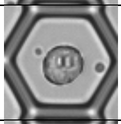	25.1	9	122 (1095)	1 (11)	9 (79)	-	-
6	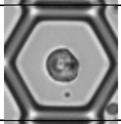	19.8	4	121 (486)	5 (19)	17 (69)	-	268 (1)
7	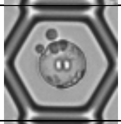	27.7	12	184 (2205)	9 (102)	10 (119)	-	292 (4)
8	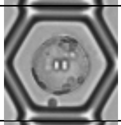	35.0	24	220 (5269)	22 (539)	8 (187)	-	161 (4)
9	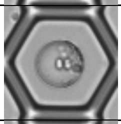	28.0	12	246 (2955)	12 (148)	9 (102)	-	171 (2)
10	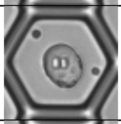	25.0	9	162 (1460)	6 (55)	11 (102)	-	273 (2)
11	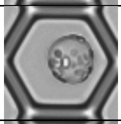	28.5	13	232 (3011)	14 (183)	10 (126)	-	176 (2)
12	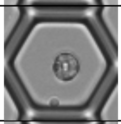	16.5	2	95 (190)	9 (18)	29 (57)	-	672 (1)
13	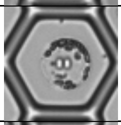	29.3	14	2 (27)	0.4 (6)	2 (31)	-	-
14	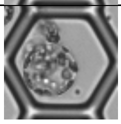	32.1	18	49 (884)	1 (23)	10 (180)	207 (4)	1400 (25)

15	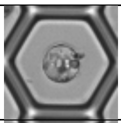	21.6	6	115 (691)	1 (3)	13 (77)	523 (3)	786 (5)
16	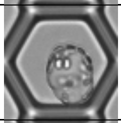	31.4	17	43 (731)	11 (181)	9 (150)	-	136 (2)
17	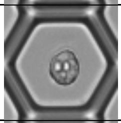	18.6	4	69 (278)	3 (13)	44 (176)	-	-
18	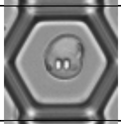	25.9	10	31 (306)	4 (42)	14 (141)	-	145 (1)
19	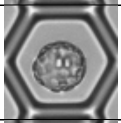	32.8	20	231 (4627)	26 (518)	15 (301)	122 (2)	1277 (26)
20	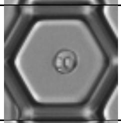	15.4	2	128 (256)	9 (17)	44 (88)	-	821 (2)
21	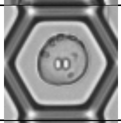	31.0	17	18 (309)	9 (154)	14 (245)	-	194 (3)
22	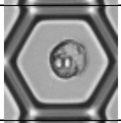	22.9	7	35 (247)	2 (12)	18 (126)	-	287 (2)
23	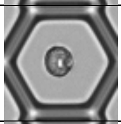	18.4	3	4 (13)	0.06 (0.2)	39 (117)	-	-
24	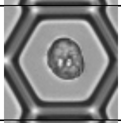	24.9	9	4 (36)	0.18 (2)	32 (292)	-	-
25	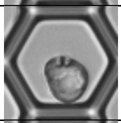	28.2	12	17 (204)	2 (25)	27 (324)	-	60 (1)
26	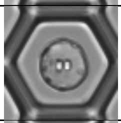	31.0	16	9 (148)	4 (66)	16 (252)	-	70 (1)
27	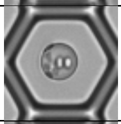	22.5	6	55 (331)	1 (6)	17 (100)	-	178 (1)
28	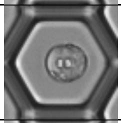	24.9	9	9 (79)	1 (5)	17 (153)	-	79 (1)
29	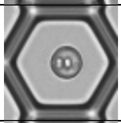	19.0	4	13 (51)	0.06 (0.3)	32 (126)	-	-
30	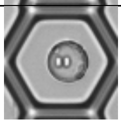	24.8	8	33 (262)	5 (39)	30 (243)	-	10 (0.08)

31	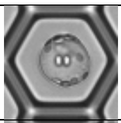	30.8	16	20 (316)	3 (45)	14 (222)	-	58 (1)
32	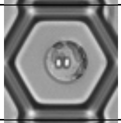	25.7	9	4 (40)	0.04 (0.4)	12 (108)	-	-
33	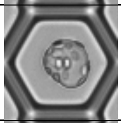	28.6	13	84 (1094)	7 (85)	12 (158)	-	268 (3)
34	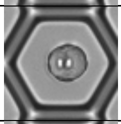	22.3	6	56 (335)	2 (10)	19 (115)	-	177 (1)
35	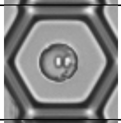	23.0	7	115 (807)	3 (19)	32 (225)	-	130 (1)
36	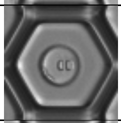	23.9	8	34 (274)	3 (22)	14 (111)	-	391 (3)
37	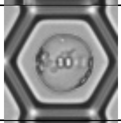	34.2	22	125 (2749)	15 (333)	18 (386)	80 (2)	592 (13)
38	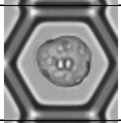	31.4	17	112 (1907)	24 (410)	14 (242)	-	323 (5)
39	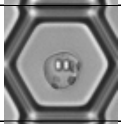	22.3	6	29 (172)	0.2 (1)	20 (120)	-	-
40	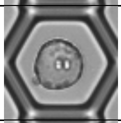	28.8	13	267 (3470)	39 (511)	16 (210)	144 (2)	757 (10)
41	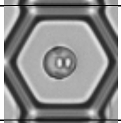	20.3	5	54 (270)	2 (8)	20 (98)	-	224 (1)
42	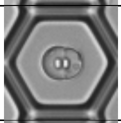	23.8	7	42 (291)	4 (25)	23 (162)	-	211 (1)
43	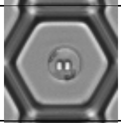	20.5	5	24 (119)	0.1 (1)	30 (151)	-	-
44	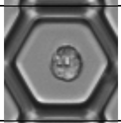	20.9	5	12 (59)	0.2 (1)	18 (92)	-	159 (1)
45	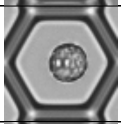	23.5	7	25 (175)	1 (8)	16 (115)	-	152 (1)
46	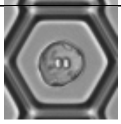	27.4	11	117 (1283)	5 (52)	19 (209)	-	97 (1)

47	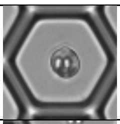	18.8	4	9 (38)	0.4 (1)	19 (75)	-	-
	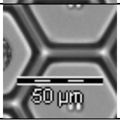	Scale<br>50 µm						
		Total number		47	47	47	5	37
		Average		90 (1031)	7 (91)	18 (154)	215 (3)	317 (4)

[a] Diameter of the protoplasts was measured by ImageJ Software.
[b] Volume of the protoplasts was calculated under the assumption that the protoplasts are spherical.
[c] The absolute amount of compounds in one protoplast (number in parentheses) was calculated using external calibration curves; the concentration of compounds in one cell was calculated by dividing the absolute amount to the volume of the protoplast.
“-“: Below the limit of quantification.

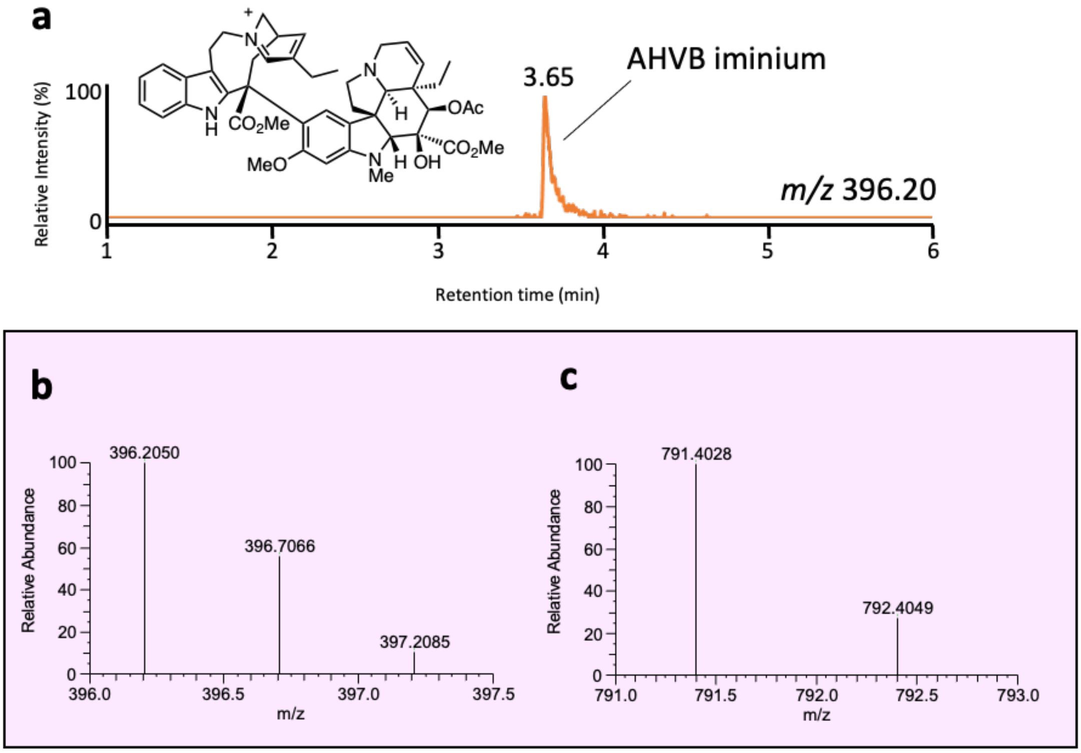

Supplementary Fig. 14: Detection of AHVB iminium in an idioblast cell. a, Extracted ion chromatogram of *m/z* 396.20. **b**, AHVB could be detected as a doubly charged ion $[M^{+}+H]^{2+}$, as evidenced by the isotopic pattern or as a singly charged species $[M^{+}]$ (**c**).

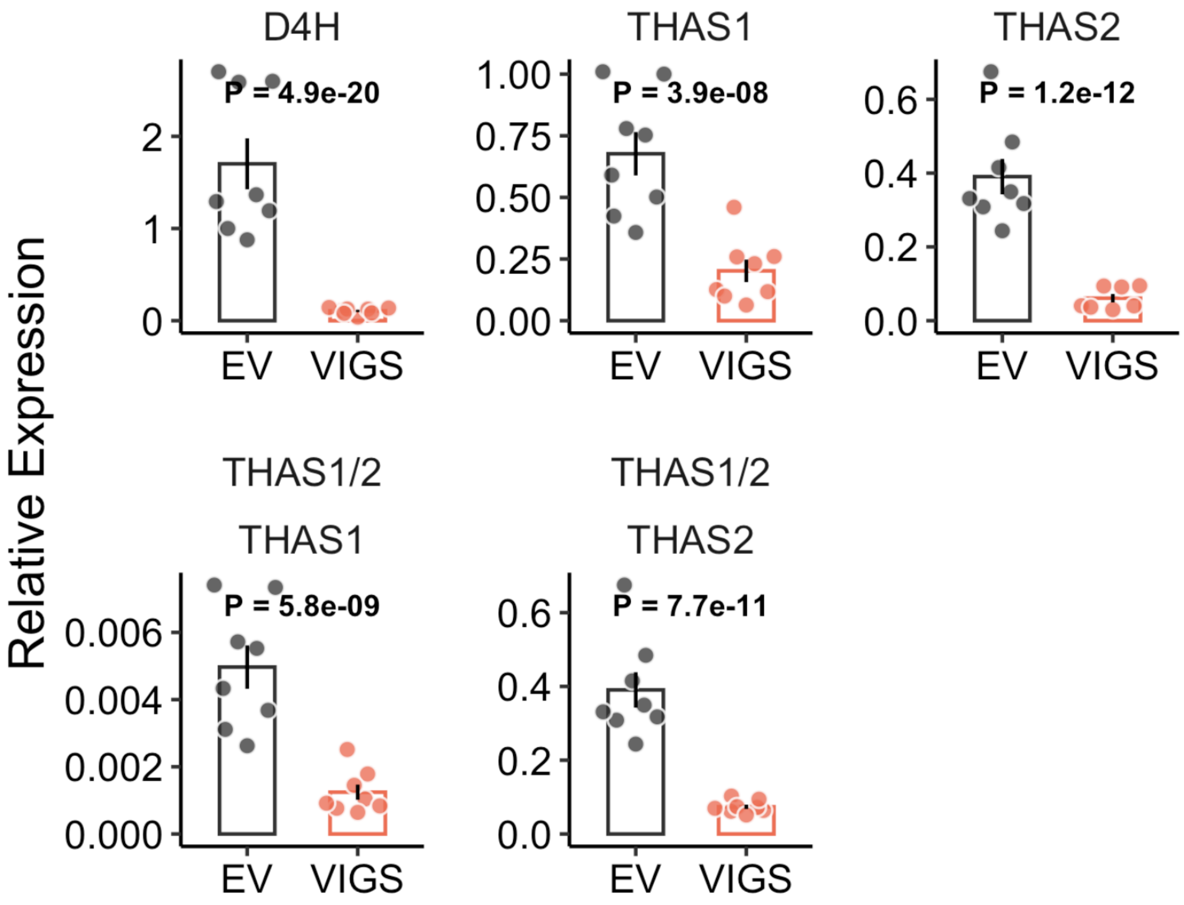

Supplementary Fig. 15: Virus-induced gene silencing of D4H, THAS1, THAS2 and of the THAS1/2 co-silencing in *C. roseus*. The graphs show the normalized relative expression of the silenced gene (VIGS) compared to the empty vector (EV) control plants as measured by qPCR. Values were calculated using 2-**ΔΔ**Ct and using Rps9 as reference gene. 8 biological replicates for control (EV) and silenced plants were used (n=8). Data are presented as mean values +/- SEM. Unpaired, two-tailed t-test was used for comparisons and the P value is shown in the graphs.

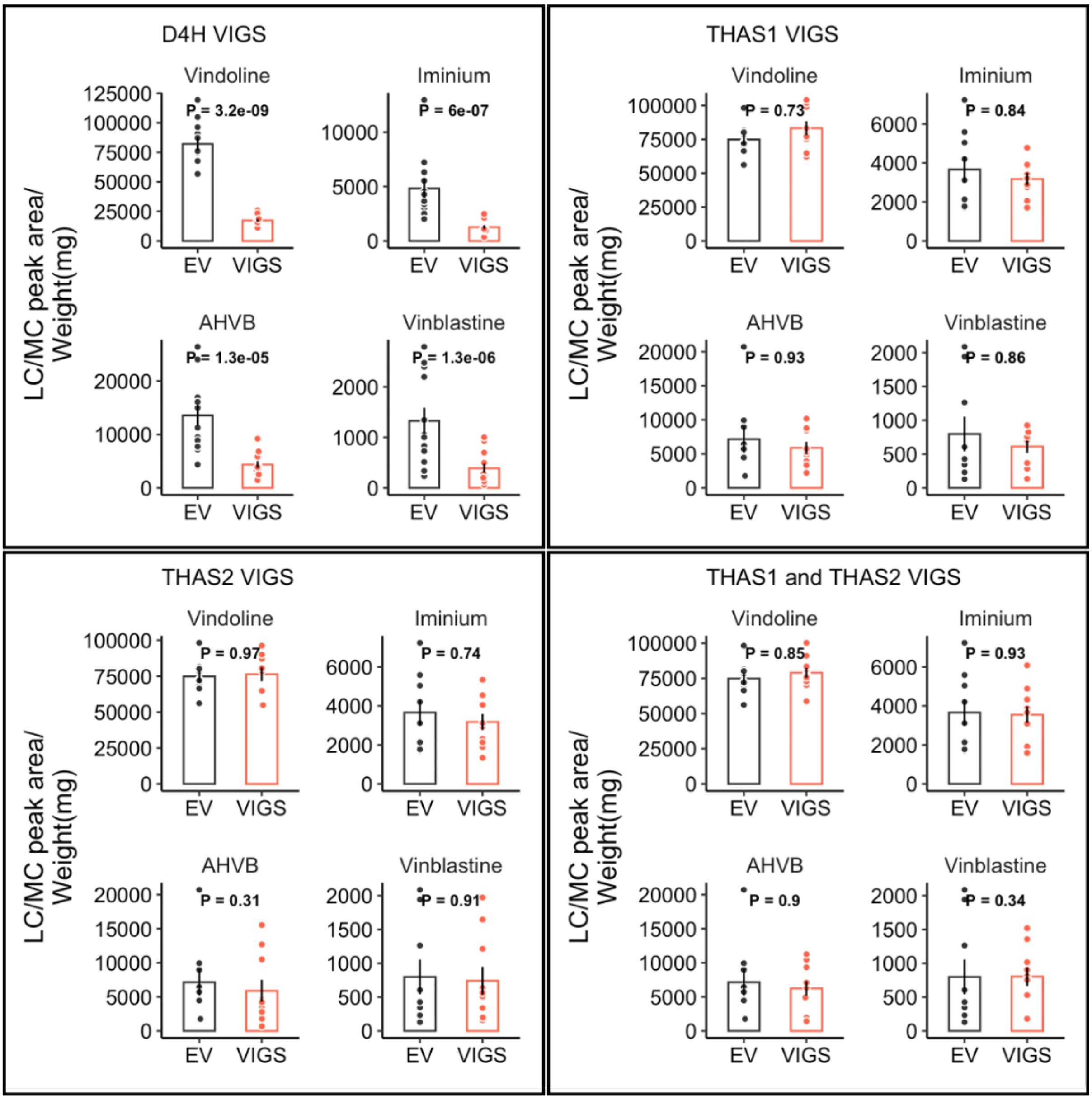

Supplementary Fig. 16: Metabolite analysis of tissue that has been transformed with a VIGS vector that targets D4H, THAS1, THAS2 and both THAS1/2 relative to the empty vector (EV) control. The graphs show the normalized peak areas of the four key metabolites vindoline, iminium dimer, vinblastine and anhydrovinblastine. 8 biological replicates for control (EV) and silenced plants were used (n=8). Data are presented as mean values +/- SEM. Unpaired, two-tailed t-test was used for comparisons and the P value is reported in the graphs.

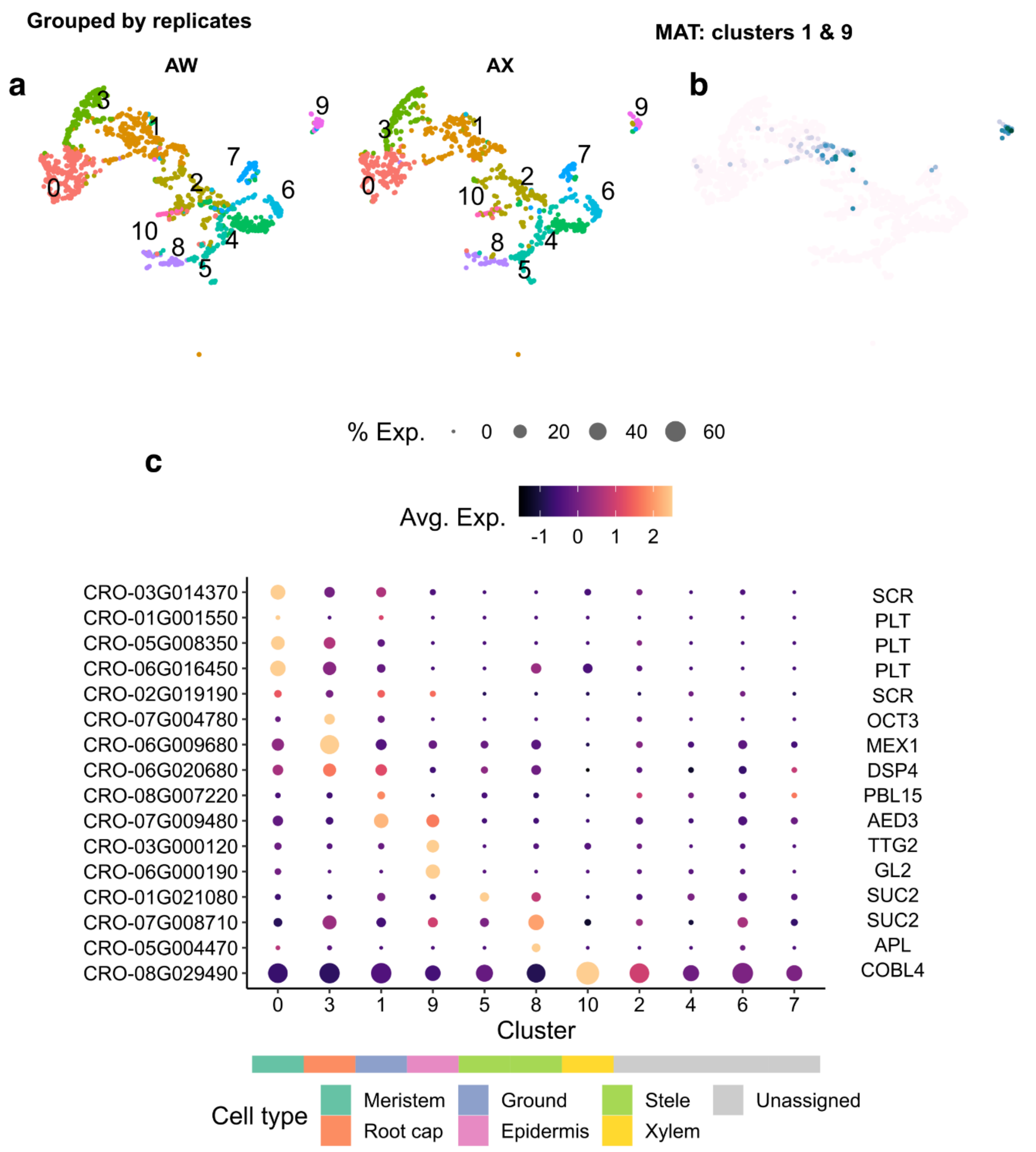

Supplementary Fig. 17: Dimension reduction and cell type assignment for root scRNA-seq data. a, UMAP (uniform manifold approximation and projection) for *C. roseus* roots; subpanels grouped by biological replicates. **b**, UMAP colored by expression of *MAT*, which is detected in Clusters 4 and 8. **c**, Cell type assignment for root scRNA-seq dataset. Each row is a marker gene (see also Supplemental Table 8). v3 annotation gene IDs are on the left; Arabidopsis ortholog gene names on the right. Dot color indicates expression level. Dot size indicates the percentage of cells expressing the marker.

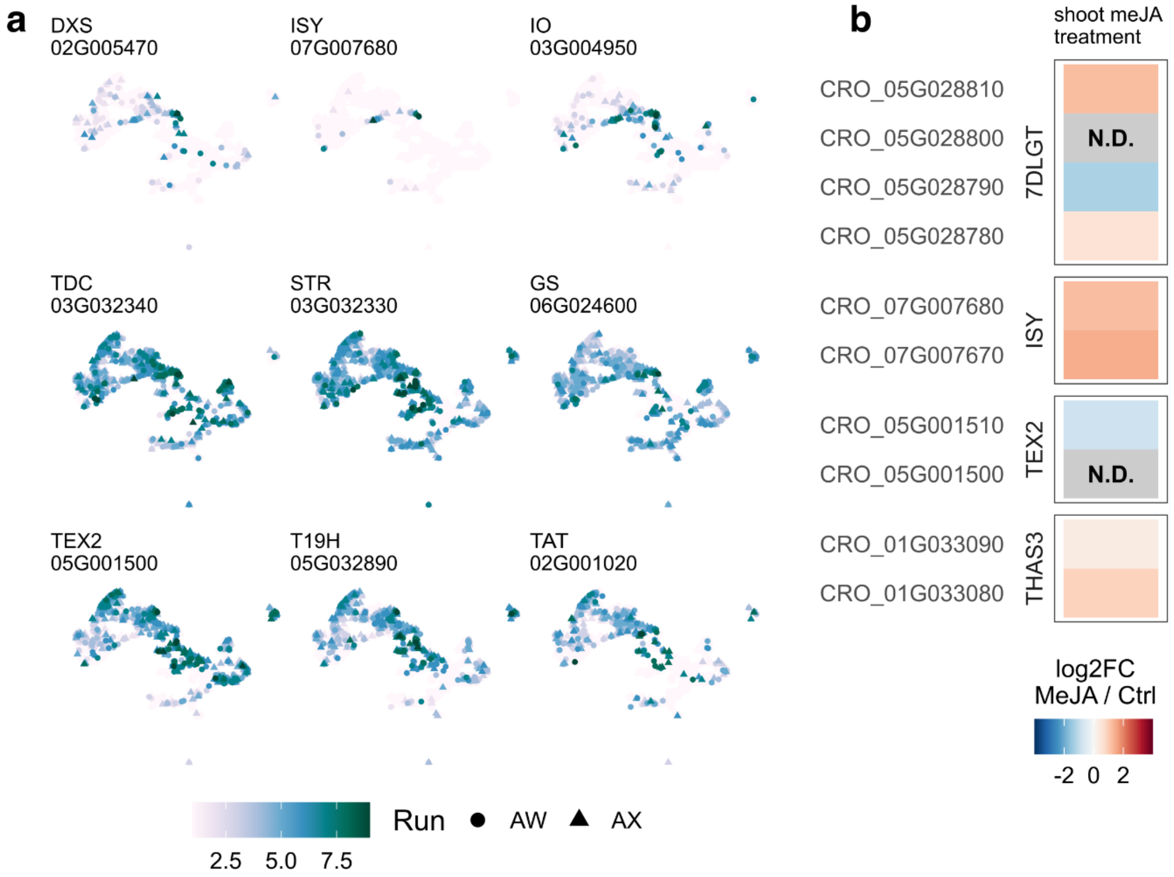

Supplementary Fig. 18: Expression of selected MIA biosynthetic genes in the root. **a**, UMAP graphs colored by expression of MIA biosynthetic genes in the root. **b**, JA-responsiveness of tandemly duplicated biosynthetic gene paralogs. Higher value (warmer color) indicates higher expression in JA-treated samples.

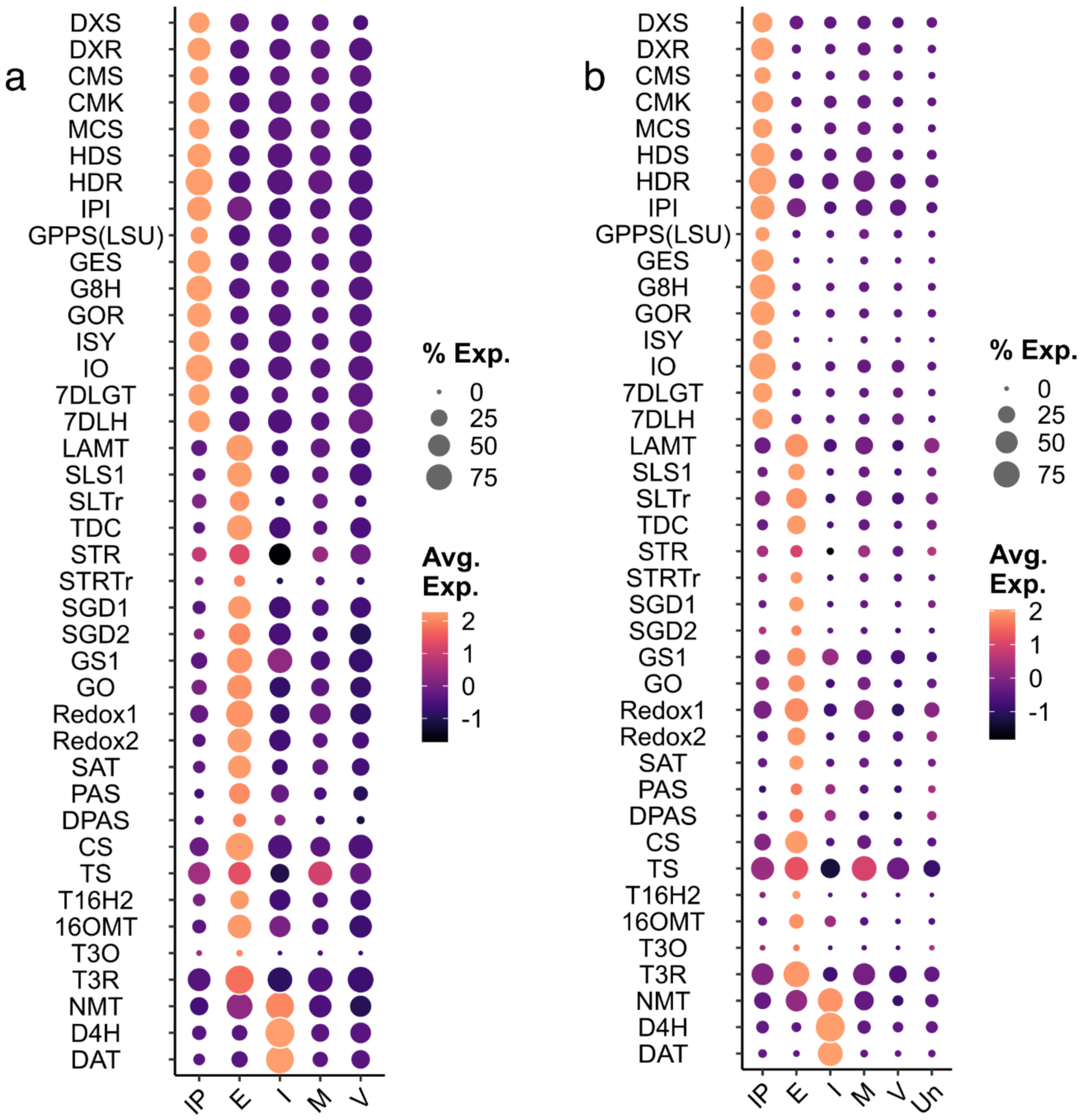

Supplementary Fig. 19: Dot plots showing expression of MIA biosynthetic gene across cell type. **a**, 10x libraries before ambient RNA removal. **b**, 10x libraries after ambient RNA removal applied to samples AT and AS, which were over-sequenced (see also Supplementary Table 11). Each row is a marker gene (see also Supplementary Table 8). Dot color indicates expression level. Dot size indicates the percentage of cells expressing the marker. IP: IPAP-internal phloem associated parenchyma; E: epidermis; I; idioblast; M: mesophyll; V: vasculature; Un: unassigned.

SUPPLEMENTARY REFERENCES

Albert, N.W. (2015). Subspecialization of R2R3-MYB Repressors for Anthocyanin and Proanthocyanidin Regulation in Forage Legumes. Front. Plant Sci. **6**: 1165.

Burlat, V., Oudin, A., Courtois, M., Rideau, M., and St-Pierre, B. (2004). Co-expression of three MEP pathway genes and geraniol 10-hydroxylase in internal phloem parenchyma of *Catharanthus roseus* implicates multicellular translocation of intermediates during the biosynthesis of monoterpene indole alkaloids and isoprenoid-derived primary metabolites. Plant J. **38**: 131–141.

De Rybel, B., Möller, B., Yoshida, S., Grabowicz, I., Barbier de Reuille, P., Boeren, S., Smith, R.S., Borst, J.W., and Weijers, D. (2013). A bHLH complex controls embryonic vascular tissue establishment and indeterminate growth in Arabidopsis. Dev. Cell **24**: 426–437.

Denyer, T., Ma, X., Klesen, S., Scacchi, E., Nieselt, K., Timmermans, and M., C.P. (2018). Spatiotemporal Developmental Trajectories in the Arabidopsis Root Revealed Using High-Throughput Single Cell RNA Sequencing. Dev. Cell **28: 840-852.e5**.

Efroni, I., Ip, P.-L., Nawy, T., Mello, A., and Birnbaum, K.D. (2015). Quantification of cell identity from single-cell gene expression profiles. Genome Biol. **16**: 9.

Jean-Baptiste, K., McFaline-Figueroa, J.L., Alexandre, C.M., Dorrity, M.W., Saunders, L., Bubb, K.L., Trapnell, C., Fields, S., Queitsch, C., and Cuperus, J.T. (2019). Dynamics of Gene Expression in Single Root Cells of *Arabidopsis thaliana*. Plant Cell **31**: 993–1011.

Kim, J.-Y. et al. (2021). Distinct identities of leaf phloem cells revealed by single cell transcriptomics. Plant Cell **33**: 511–530.

Miettinen, K. et al. (2014). The seco-iridoid pathway from *Catharanthus roseus*. Nat. Commun. **5**: 3606.

Ohashi-Ito, K. and Bergmann, D.C. (2006). Arabidopsis FAMA controls the final proliferation/differentiation switch during stomatal development. Plant Cell **18**: 2493–2505.

Ryu, K.H., Huang, L., Kang, H.M., and Schiefelbein, J. (2019). Single-Cell RNA Sequencing Resolves Molecular Relationships Among Individual Plant Cells. Plant Physiol. **179**: 1444–1456.

Shulse, C.N., Cole, B.J., Ciobanu, D., Lin, J., Yoshinaga, Y., Gouran, M., Turco, G.M., Zhu, Y., O'Malley, R.C., Brady, S.M., and Dickel, D.E. (2019). High-Throughput Single-Cell Transcriptome Profiling of Plant Cell Types. Cell Rep. **27**: 2241-2247.e4.

Tsugeki, R. and Fedoroff, N.V. (1999). Genetic ablation of root cap cells in Arabidopsis. Proc. Natl. Acad. Sci. U. S. A. **96**: 12941–12946.
