# Supplementary material for: Single-cell multi-omics in the medicinal plant Catharanthus roseus
Source: Nat Chem Biol. 2023 May 15;19(8):1031–41. doi: 10.1038/s41589-023-01327-0 (PMC10374443; doi:10.1038/s41589-023-01327-0)
Supplement: Supplementary file 2 — Reporting Summary [file 41589_2023_1327_MOESM2_ESM.pdf]

nature research

Corresponding author(s): Buell, O'Connor, Caputi

Last updated by author(s): Feb 20, 2023

Reporting Summary

Nature Research wishes to improve the reproducibility of the work that we publish. This form provides structure for consistency and transparency in reporting. For further information on Nature Research policies, see our Editorial Policies and the Editorial Policy Checklist.

Statistics

For all statistical analyses, confirm that the following items are present in the figure legend, table legend, main text, or Methods section.

n/a	Confirmed	
☐	☒	The exact sample size (*n*) for each experimental group/condition, given as a discrete number and unit of measurement
☐	☒	A statement on whether measurements were taken from distinct samples or whether the same sample was measured repeatedly
☐	☒	The statistical test(s) used AND whether they are one- or two-sided<br>*Only common tests should be described solely by name; describe more complex techniques in the Methods section.*
☒	☐	A description of all covariates tested
☐	☒	A description of any assumptions or corrections, such as tests of normality and adjustment for multiple comparisons
☐	☒	A full description of the statistical parameters including central tendency (e.g. means) or other basic estimates (e.g. regression coefficient) AND variation (e.g. standard deviation) or associated estimates of uncertainty (e.g. confidence intervals)
☐	☒	For null hypothesis testing, the test statistic (e.g. *F*, *t*, *r*) with confidence intervals, effect sizes, degrees of freedom and *P* value noted<br>*Give P values as exact values whenever suitable.*
☒	☐	For Bayesian analysis, information on the choice of priors and Markov chain Monte Carlo settings
☒	☐	For hierarchical and complex designs, identification of the appropriate level for tests and full reporting of outcomes
☒	☐	Estimates of effect sizes (e.g. Cohen's *d*, Pearson's *r*), indicating how they were calculated

Our web collection on statistics for biologists contains articles on many of the points above.

Software and code

Policy information about availability of computer code

Data collection	Xcalibur 4.3.73.11: mass spectrometry acquisition
Data analysis	3D-DNA (git commit 429ccf4): Used for genome scaffolding<br>BRAKER (v2.1.5): Used for genome annotation<br>CAMERA (v1.54.0): mass spectrometry analysis<br>Cufflinks (v2.2.1): Gene expression abundance estimation<br>Cutadapt (v2.10; v4.0): Read cleaning<br>DENTIST (v3.0.0): Assembly gap filling<br>DropSeqTools (v2.5.1): Single cell RNA seq<br>Flye (v2.8.3-b1695): Genome assembler<br>Guppy (5.0.7+2332e8d; v5.0.16; v6.0.6+8a98bbc): Base caller<br>HiCCUPS (v1.19.01): Chromosome loop finder<br>HiCkey (v1.0): TAD boundary finder<br>HISAT2 (v2.1.0, v2.2.1,): Read aligner<br>Juicer (v1.6): Software for Hi-C data<br>Juicer Tools (v2.13.07): Software for Hi-C data<br>Medaka (v1.4.3): Error correction for genome assemblies<br>PASA2 (v2.4.1): Gene model refinement<br>Pilon (v1.23): Error correction for genome assemblies<br>ProtExcluder (v1.2): Identifies non-transposable element genes from libraries<br>Pychopper (v2.5.0): Processes Oxford Nanopore cDNA reads<br>R (v4.1.3): General programming language<br>Racon (v1.4.20): Error correction

RepeatMasker (v4.1.2): Genome assembly
RepeatModeler (v2.0.3): De novo predictor of repetitive sequences
seqtk (v1.3): Sequence software
Seurat (v3): Single cell analysis software
STARsolo (v2.7.10): Read alignment software
Straw (v1.0): Extract data from .HiC objects
StringTie2 (v2.2.1): Transcript assembly software
WebApollo (v2.6.5): Gene model annotation software
XCMS centWave (v3.20.0): Peak annotation
DecontX (v1.14.2): removal of ambient RNA reads from single cell RNA-seq datasets
Custom R scripts for data visualization: https://github.com/cxli233/Catharanthus_scRNA_seq

For manuscripts utilizing custom algorithms or software that are central to the research but not yet described in published literature, software must be made available to editors and reviewers. We strongly encourage code deposition in a community repository (e.g. GitHub). See the Nature Research guidelines for submitting code & software for further information.

Data

Policy information about availability of data

All manuscripts must include a data availability statement. This statement should provide the following information, where applicable:

- Accession codes, unique identifiers, or web links for publicly available datasets
- A list of figures that have associated raw data
- A description of any restrictions on data availability

Data supporting the findings of this work are available within the paper and its Supplementary Information files. Sequences of the genes THAS1 (KM524258.1), THAS2 (KU865323.1), ADH20 (KU865330.1), ADH32 (AYE56096.1), and ADH92 (ON911573) are available from Genbank. All sequencing data associated with this study are available at the National Center for Biotechnology Institute Short Read Archive BioProject PRJNA847226. Large files including the gene expression abundances from the bulk mRNA-seq, leaf and root scRNA-seq, genome assembly and genome annotation are available via the Dryad Digital Repository. A reporting summary for this Article is available as a Supplementary Information file.

Field-specific reporting

Please select the one below that is the best fit for your research. If you are not sure, read the appropriate sections before making your selection.

☒ Life sciences ☐ Behavioural & social sciences ☐ Ecological, evolutionary & environmental sciences

For a reference copy of the document with all sections, see nature.com/documents/nr-reporting-summary-flat.pdf

Life sciences study design

All studies must disclose on these points even when the disclosure is negative.

Sample size	For generation of genome assembly, only a single accession was used. For scRNA-seq expression abundances, multiple plants were collated into replicates as reported in the Methods. TAD analyses was generated from pooled tissue; a single library was constructed. For VIGS studies, 8 biological replicates for control (EV) and silenced plants were used. This sample size for this type of experiment was validated in the original publication of the method (Liscombe & O'Connor, 2011). Unpaired, two-tailed Tukey tests were used for comparisons. Protein activity assays were performed in triplicates, as the results were tightly distributed. No other sample size calculations were done. Levels of replications for sequencing experiments are consistent with the literature.
Data exclusions	For single cell experiments, empty droplets and multiplets were discarded based on number of UMI and number of genes expressed (see Methods). No other data were excluded.
Replication	For scRNAseq expression analyses, biological replicate reproducibility was assessed and reported in Materials and Results section.
Randomization	Samples for gene expression profiling were randomized in the growth chamber. For enzyme assays, samples were loaded randomly on the mass spectrometer. Blinding is not performed for sequencing or biochemical experiments since blinding is incompatible with the experimental process. For the single cell metabolomics experiment, the cells were collected and prepared for analysis in 96-well plates. The order of the injections into the LC/MS system were randomized to reduce variance. Randomization was performed using the RAND function in Excel. LC/MS analysis of the enzyme activity assays and VIGS samples were also performed in a randomized way.
Blinding	Blinding is not performed for sequencing or biochemical experiments since blinding is incompatible with the experimental process. The experiments required that the experimenters be aware of the identities of the treatments and tissues.

Reporting for specific materials, systems and methods

We require information from authors about some types of materials, experimental systems and methods used in many studies. Here, indicate whether each material, system or method listed is relevant to your study. If you are not sure if a list item applies to your research, read the appropriate section before selecting a response.

Materials & experimental systems

n/a	Involved in the study
☒	☐ Antibodies
☒	☐ Eukaryotic cell lines
☒	☐ Palaeontology and archaeology
☒	☐ Animals and other organisms
☒	☐ Human research participants
☒	☐ Clinical data
☒	☐ Dual use research of concern

Methods

n/a	Involved in the study
☒	☐ ChIP-seq
☒	☐ Flow cytometry
☒	☐ MRI-based neuroimaging
